# Mitochondrial Dysfunction in Metabolic-Syndrome-Related MASLD/MASH: Metabolic Mechanisms and Therapeutic Perspectives

**DOI:** 10.3390/metabo16070489

**Published:** 2026-07-11

**Authors:** Jin Jin, Yang Cheng

**Affiliations:** Institute of Liver Diseases, Shuguang Hospital Affiliated to Shanghai University of Traditional Chinese Medicine, Shanghai 201203, China

**Keywords:** MASLD, MASH, mitochondrial dysfunction, metabolic syndrome, insulin resistance, oxidative stress, mitophagy, mitochondrial quality control, metabolomics, fibrosis

## Abstract

**Highlights:**

**What are the main findings?**

- Mitochondrial dysfunction links lipid overload to impaired fatty acid oxidation, oxidative stress, ER-mitochondria crosstalk, defective mitophagy, and mitochondrial danger-signal release.
- Mitochondrial danger signals connect hepatocyte injury with macrophage activation, hepatic stellate cell activation, extracellular matrix deposition, and fibrosis.

**What are the implications of the main findings?**

- Therapeutic strategies for metabolic-syndrome-related MASLD/MASH should be organized by evidence level into upstream metabolic unloading, midstream mitochondrial restoration, and downstream anti-inflammatory or antifibrotic intervention.
- Metabolomics and mitochondrial readouts should be interpreted as mechanistic and stage-dependent tools rather than stand-alone diagnostic markers.

**Abstract:**

Background/Objectives: Metabolic-dysfunction-associated steatotic liver disease (MASLD) and metabolic-dysfunction-associated steatohepatitis (MASH) arise in the setting of obesity, insulin resistance, type 2 diabetes, and metabolic syndrome. This review examines how mitochondrial dysfunction participates in the transition from lipid accumulation to hepatocyte injury, inflammation, and fibrosis, and how evidence from human, animal, and in vitro studies should be interpreted. Methods: We provide a narrative synthesis of mechanistic, translational, and clinical studies on hepatic mitochondrial metabolism, fatty acid oxidation, oxidative phosphorylation, redox stress, organelle crosstalk, mitophagy, mitochondrial biogenesis and proteostasis, mitochondrial danger signals, the gut-liver-mitochondria axis, and mitochondria-related therapeutic strategies. Results: In early metabolic overload, mitochondrial oxidation may increase as an adaptive response. With persistent substrate pressure, this adaptation can become inefficient, with impaired fatty acid disposal, less efficient oxidative phosphorylation, reactive oxygen species production, redox imbalance, defective mitochondrial quality control, altered mitochondrial biogenesis, mitochondrial unfolded protein response (UPRmt)-related proteostatic stress and mtDNA instability. Mitochondrial DNA and RNA released from damaged organelles may also activate cyclic GMP-AMP synthase-stimulator of interferon genes (cGAS-STING), inflammasome, and RNA-sensing pathways, linking hepatocyte stress to macrophage activation, stellate cell activation, extracellular matrix deposition, and fibrosis. Conclusions: The current evidence supports mitochondria as a stage-dependent amplifier of metabolic liver injury rather than a uniform initiating event. Clinically, the strongest evidence remains with upstream metabolic unloading and liver-directed metabolic therapy, whereas direct mitochondrial restoration and quality-control targeting remain promising but less mature.

## 1. Introduction

### 1.1. MASLD/MASH as a Metabolic Liver Disease

Metabolic-dysfunction-associated steatotic liver disease (MASLD) can be viewed as a liver-centered manifestation of obesity, insulin resistance, type 2 diabetes, and metabolic syndrome. Its pathological spectrum ranges from simple steatosis to metabolic-dysfunction-associated steatohepatitis (MASH), fibrosis, cirrhosis, and liver-related complications in a subset of patients [1,2,3].

This progression is not explained only by the amount of fat stored in hepatocytes. It also depends on whether hepatocytes can adapt to chronic lipid and nutrient excess. When this adaptive capacity fails, lipotoxicity, oxidative injury, ER stress, and cell damage become more likely. These changes support the shift from steatosis toward inflammation and fibrotic remodeling [3,4,5].

### 1.2. Epidemiological and Clinical Burden of MASLD/MASH

MASLD/MASH arises against a backdrop of metabolic dysfunction, including obesity, insulin resistance, type 2 diabetes, and metabolic syndrome. Global estimates suggest that approximately 25–30% of adults are affected, with considerable regional variation (13% in Africa vs. 31% in the Middle East) [6]. The prevalence is higher among individuals with metabolic risk factors: 70% of people with obesity and 55–60% of those with type 2 diabetes have MASLD/NAFLD [7,8,9]. Pediatric and adolescent populations show a rising prevalence, paralleling increases in obesity and insulin resistance [10].

Progression to MASH, fibrosis, and cirrhosis occurs in a subset of patients. Around 20% of patients with MASH may develop cirrhosis, while the annual incidence of hepatocellular carcinoma (HCC) in NASH-related cirrhosis ranges from 0.5% to 2.6% [11,12]. Non-cirrhotic MASLD also carries HCC risk, albeit lower (0.1–1.3 per 1000 patient-years) [11]. Fibrosis stage remains the strongest predictor of liver-related morbidity and mortality, whereas cardiovascular disease and extrahepatic malignancies contribute substantially to overall mortality [8,9].

These epidemiological patterns highlight the clinical and societal burden of MASLD/MASH. Understanding disease progression is crucial, as only a fraction of patients advance from steatosis to MASH, fibrosis, cirrhosis, and HCC. Progression reflects complex interactions among hepatocyte lipid handling, oxidative stress, inflammation, and fibrogenic signaling rather than hepatic fat quantity alone. Clarifying these mechanisms will aid in risk stratification and guide the development of stage-specific biomarkers and therapeutic interventions [3,7].

### 1.3. Terminology and Interpretation of NAFLD/NASH-Based Evidence

This review adopts the current terminology of metabolic-dysfunction-associated steatotic liver disease (MASLD) and metabolic-dysfunction-associated steatohepatitis (MASH), emphasizing the central role of metabolic dysfunction in disease pathogenesis [12]. However, the majority of historical epidemiological, mechanistic, and therapeutic studies were conducted under the previous nomenclature of nonalcoholic fatty liver disease (NAFLD) and nonalcoholic steatohepatitis (NASH) [7,8,13,14]. These studies remain valuable because they characterize largely overlapping clinical phenotypes, including hepatic steatosis, steatohepatitis, fibrosis, cirrhosis, and hepatocellular carcinoma, often in the context of obesity, insulin resistance, type 2 diabetes, or metabolic syndrome [15].

While NAFLD/NASH evidence is informative, the new MASLD/MASH nomenclature introduces a positive diagnostic framework based on metabolic dysfunction rather than the exclusionary criteria used in NAFLD. Consequently, previous studies may differ in alcohol thresholds, definitions of metabolic risk, or inclusion of comorbidities, potentially affecting prevalence estimates, progression rates, and therapeutic responses [16].

In this review, MASLD/MASH terminology is used consistently, while older NAFLD/NASH studies are cited when they provide relevant mechanistic, epidemiological, or clinical insights. Such data are interpreted within a metabolic-dysfunction-centered framework, acknowledging the conceptual refinement introduced by the updated nomenclature. This approach ensures both the integrity of the historical evidence base and alignment with the current understanding of MASLD/MASH pathophysiology [12,13].

### 1.4. Mitochondria as a Metabolic Amplifier in Disease Progression

Mitochondria are often discussed as energy-producing organelles, but in the liver they also function as metabolic decision points. They support fatty acid β-oxidation, the tricarboxylic acid cycle, oxidative phosphorylation, ATP production, and redox balance. Through these pathways, hepatocytes decide whether incoming substrates are oxidized, stored, exported, or converted into more reactive lipid species [17,18,19].

This role becomes especially important in MASLD/MASH. During early nutrient excess, increased mitochondrial oxidation may help hepatocytes cope with a larger fatty acid load. That response is not necessarily harmful at first. It can be viewed as an adaptive compensatory phase in which hepatocytes attempt to maintain energy production and limit lipid retention. The problem appears when sustained metabolic pressure exceeds this adaptive capacity. In that setting, mitochondrial respiration becomes less efficient, ROS generation increases, ER–mitochondria stress intensifies, and mitochondrial quality control begins to decline [17,20,21].

For this review, mitochondrial dysfunction is therefore considered as a stage-dependent amplifier rather than as one isolated lesion. The progression can be summarized as a connected sequence: lipid overload first induces adaptive mitochondrial compensation; persistent substrate pressure then promotes ROS production and ER stress; defective mitophagy allows damaged mitochondria to accumulate; and mitochondrial DNA or RNA released from injured organelles can act as danger signals that activate innate immune pathways. These events link hepatocyte metabolic stress to inflammation, stellate cell activation, extracellular matrix deposition, and fibrosis.

**Figure 1:**
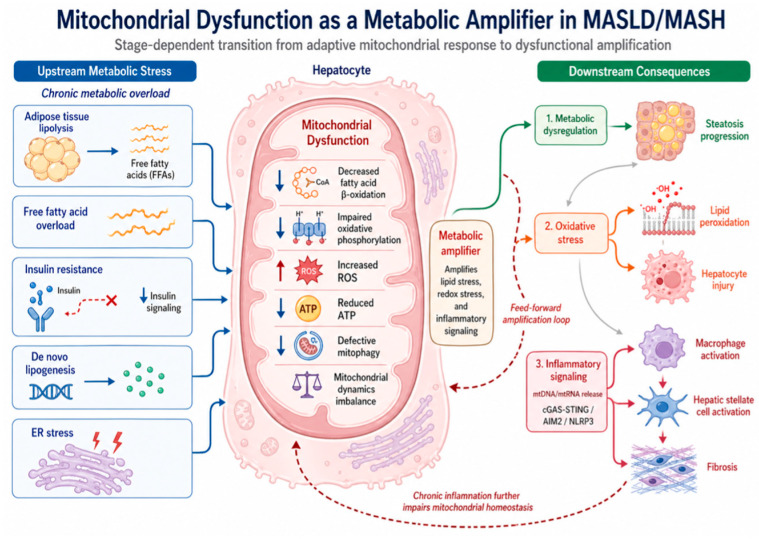
Mitochondrial dysfunction as a metabolic amplifier in MASLD/MASH.

This framing allows lipid overload, adaptive compensation, redox imbalance, ER stress, mitophagy failure, mitochondrial danger-signal release, innate immune activation, and fibrosis to be discussed as connected events in disease progression (Figure 1).

### 1.5. Novelty and Conceptual Framework of This Review

This review is not a general summary of mitochondrial dysfunction in MASLD/MASH. Its novelty lies in integrating metabolic context, disease stage, and translational relevance into a coherent framework.

First, we focus on metabolic-syndrome-related MASLD/MASH, emphasizing how obesity, insulin resistance, type 2 diabetes, and dyslipidemia drive hepatic mitochondrial stress and disease progression [12,13]. This approach differs from prior reviews that often considered all NAFLD cases indiscriminately.

Second, we adopt a stage-dependent perspective. Early mitochondrial responses to nutrient overload may be adaptive, supporting fatty acid oxidation and energy homeostasis. With persistent metabolic pressure, these responses can become pathological, characterized by inefficient respiration, ROS and ER stress, mitophagy failure, and mitochondrial danger-signal release, which in turn promote inflammation and fibrosis [17,22]. Distinguishing adaptive remodeling from pathological dysfunction clarifies the timing and role of mitochondrial changes in disease progression.

Third, we separate human, animal, and in vitro evidence. Human and translational studies are prioritized for disease phenotype, fibrosis stage, metabolic readouts, and therapeutic response, whereas animal and cell studies are used to explore mechanisms not directly accessible in patients, such as ER–mitochondria crosstalk and mitophagy failure [7,14].

Finally, this review links mitochondrial mechanisms to biomarkers and therapy. Metabolomic and lipidomic readouts, mitochondrial quality-control markers, and oxidative stress indicators are interpreted in a stage-dependent manner, highlighting opportunities for early detection and stage-specific intervention. Therapeutic strategies are organized according to metabolic, mitochondrial, and downstream inflammatory or fibrotic targets [7,13].

Collectively, these features provide a framework that connects metabolic overload, mitochondrial stress, hepatocyte injury, and fibrosis while emphasizing disease stage, evidence type, and translational relevance.

To clarify how different evidence sources are interpreted in this review, we summarize human, animal, and in vitro evidence for major mitochondrial mechanisms in Table 1. This table is intended to prevent overinterpretation of preclinical findings and to distinguish clinically supported observations from mechanistic hypotheses.

### 1.6. Review Strategy and Organization

This narrative review brings together studies that link mitochondrial stress to the progression of MASLD/MASH. We gave priority to human and translational work when it was available and used cell or animal studies mainly to clarify mechanisms that are difficult to test directly in patients. The review is not a systematic review and does not include formal meta-analysis. Section 2 summarizes normal hepatic mitochondrial functions. Section 3 then follows the disease process from lipid overload and impaired fatty acid oxidation to oxidative stress, ER-mitochondria crosstalk, mitochondrial quality-control failure, inflammation, and fibrosis. Section 4 considers metabolic readouts, therapeutic implications, and current limitations. Section 5 summarizes the main conclusions and discusses mitochondrial outer membrane permeabilization (MOMP) as a cautious future direction.

## 2. Mitochondria as a Metabolic Hub in the Liver

### 2.1. Hepatocyte-Specific Mitochondrial Metabolism

Hepatocyte mitochondria should not be viewed only as ATP-producing organelles. In the liver, they support a broader set of metabolic tasks that are central to whole-body nutrient homeostasis. These include fatty acid β-oxidation, ketone body production, pyruvate oxidation, TCA cycle activity, redox balance [23], and the mitochondrial steps that support gluconeogenesis [24], amino acid catabolism, and nitrogen disposal [25].

This hepatocyte-specific role is most evident during feeding and fasting. In the fed state, insulin favors glycolysis and lipogenesis, and hepatocytes channel excess carbon into fatty acid and triglyceride synthesis, lipid droplet storage, membrane lipids, or VLDL secretion [23]. In the fasted state, hepatic metabolism shifts toward glucose production through glycogenolysis and gluconeogenesis. With prolonged fasting, fatty acids released from adipose tissue are delivered to the liver, where mitochondrial β-oxidation provides acetyl-CoA and reducing equivalents for energy metabolism and ketogenesis. The resulting ketone bodies are exported and used as metabolic fuel by extrahepatic tissues [23].

Mitochondria also support hepatic glucose production. Pyruvate carboxylation, mitochondrial oxaloacetate handling, malate/aspartate shuttling, and redox exchange help connect mitochondrial carbon metabolism to cytosolic gluconeogenic flux. This connection means that hepatic gluconeogenesis is not simply a cytosolic pathway. It depends on mitochondrial substrate handling, amino-acid-derived carbon input, and the coordination between mitochondrial and cytosolic compartments [24].

Nitrogen metabolism is another defining feature of hepatocyte mitochondrial function. Hepatic mitochondria participate in amino acid catabolism and ammonia detoxification through urea-cycle-related metabolism [25]. The hepatic urea cycle is the principal pathway for systemic ammonia detoxification, and reduced urea cycle activity has been discussed as a possible contributor to NAFLD/MASLD pathophysiology [25,26]. These functions distinguish hepatocytes from many other cell types because mitochondrial metabolism in the liver must coordinate carbon flux with nitrogen disposal.

Hepatocyte mitochondria are also linked to lipid and bile acid metabolism. Fatty acids entering the liver can be oxidized, stored, transformed, or secreted, and this partitioning is central to whether hepatocytes remain metabolically flexible or accumulate lipid [27]. Bile acid signaling through hepatic nuclear receptor pathways further connects cholesterol, triglyceride, glucose, and bile acid metabolism [28]. Thus, mitochondrial metabolism operates within a larger hepatocyte network that integrates lipid handling, bile-acid-related signaling, and systemic metabolic adaptation.

This role differs from mitochondria in highly energy-demanding tissues such as the heart, skeletal muscle, and neurons. Cardiac mitochondria are specialized to sustain continuous ATP supply for contraction [29], skeletal muscle mitochondria adapt strongly to exercise and oxidative demand [30], and neuronal mitochondria support ATP production together with calcium handling, ROS signaling, and neuronal development [31]. Hepatocyte mitochondria certainly produce ATP, but they are better understood as metabolic allocation centers that decide how carbon, lipid, nitrogen, and redox equivalents are distributed across competing pathways.

This hepatocyte-specific background is important for MASLD/MASH. In metabolic overload, the problem is not only that mitochondria fail to generate ATP. Rather, chronic nutrient excess disrupts the ability of hepatocyte mitochondria to distribute substrates safely among oxidation, ketogenesis, gluconeogenesis, lipid storage, secretion, nitrogen disposal, and stress signaling. Once this metabolic flexibility is lost, mitochondrial dysfunction can amplify lipid retention, oxidative stress, inflammatory signaling, and fibrotic remodeling.

### 2.2. Fatty Acid β-Oxidation, Ketogenesis, and Lipid Fate

The liver receives fatty acids from the diet, adipose tissue lipolysis, and de novo lipogenesis. Hepatocytes therefore depend on mitochondrial fatty acid β-oxidation to process a large and variable lipid load. This pathway converts fatty acyl chains into acetyl-CoA, NADH, and FADH_2_, which then support the TCA cycle and oxidative phosphorylation [4,17,32].

Fatty acid oxidation limits hepatic lipid retention. When this pathway is efficient, incoming fatty acids can be used for energy production rather than being redirected into triglyceride storage or toxic lipid intermediates. During fasting, hepatic β-oxidation also provides acetyl-CoA for ketone body production, which shows that mitochondrial lipid oxidation is a normal adaptive pathway as well as a defense against lipid overload [33,34].

A useful way to frame this process is lipid fate. After fatty acids enter hepatocytes, they can be oxidized in mitochondria, esterified and stored as triglycerides in lipid droplets, packaged and exported as VLDL, or converted into bioactive and potentially lipotoxic intermediates [27,35]. Hepatic fat accumulation therefore reflects not only increased lipid input, but also how hepatocytes distribute fatty acids among oxidation, storage, secretion, and lipotoxic conversion.

In this sense, hepatocyte mitochondria help determine lipid fate. Efficient mitochondrial oxidation reduces the pool of fatty acids available for triglyceride accumulation or conversion into lipotoxic species. By contrast, when mitochondrial fatty acid handling becomes insufficient or inefficient, fatty acids are more likely to remain in the cytosol, expand lipid droplets, enter secretion or storage pathways, or generate lipid intermediates that disturb insulin signaling, ER homeostasis, mitochondrial function, and inflammatory responses [35,36].

This background matters for MASLD/MASH because hepatic fat accumulation reflects both lipid supply and mitochondrial disposal capacity. If fatty acid oxidation falls, fatty acids are no longer processed efficiently in mitochondria. They are then more likely to remain in the cytosol, expand lipid droplets, or enter lipotoxic pathways. This point is developed in Section 3, where impaired oxidation is discussed together with lipotoxic injury.

### 2.3. Electron Transport Chain, Cytochrome System, and ATP Synthesis

Oxidative phosphorylation links mitochondrial substrate oxidation to ATP production through the electron transport chain (ETC). In hepatocyte mitochondria, reducing equivalents generated from fatty acid β-oxidation, the TCA cycle, and other mitochondrial pathways enter the ETC mainly as NADH and FADH_2_. These electrons are transferred through complexes I–IV, while complex V uses the resulting proton gradient to synthesize ATP [37,38].

Complex I accepts electrons from NADH and transfers them to ubiquinone. Complex II receives electrons from succinate oxidation and also delivers them to ubiquinone, although it does not pump protons across the inner mitochondrial membrane. Reduced ubiquinone then carries electrons to complex III. From complex III, electrons are transferred to cytochrome c, a small soluble protein in the intermembrane space. Cytochrome c then delivers electrons to complex IV, also known as cytochrome c oxidase, where oxygen is reduced to water [37,39].

This cytochrome system is central to mitochondrial respiration. Cytochrome c acts as the mobile electron carrier between complex III and complex IV, whereas complex IV/cytochrome c oxidase is the terminal oxidase of the ETC. By accepting electrons from cytochrome c and reducing molecular oxygen, complex IV helps maintain electron flow through the respiratory chain and contributes to the proton-motive force that drives ATP synthesis [39,40].

Electron transport is not perfectly efficient. A small fraction of electrons can leak from the respiratory chain and react with oxygen to form superoxide. Complexes I and III are commonly discussed as major ETC-related sites of electron leak and mitochondrial ROS generation. In MASLD/MASH, lipid overload and altered mitochondrial substrate supply can increase pressure on the respiratory chain. Under these conditions, preserved or increased fatty acid oxidation may coexist with less efficient ETC function, favoring ROS production upstream of cytochrome c oxidase [38,41].

ROS generation is therefore closely linked to ETC function. At physiological levels, mitochondrial ROS can participate in redox signaling. When ROS production exceeds antioxidant capacity, however, oxidative stress can damage mitochondrial DNA, proteins, and membrane lipids, further impairing respiratory-chain function. This creates a feed-forward loop in which ETC dysfunction increases ROS generation, and ROS-mediated damage further compromises oxidative phosphorylation [38,41].

Complex V, or ATP synthase, converts the proton-motive force into ATP. As complexes I, III, and IV transfer electrons, they pump protons from the matrix to the intermembrane space. This establishes an electrochemical gradient across the inner mitochondrial membrane. Protons then flow back into the matrix through ATP synthase, driving the phosphorylation of ADP to ATP. Thus, ATP synthesis depends not only on substrate availability, but also on coordinated electron flow, proton pumping, inner-membrane integrity, and ATP synthase activity [37,40].

In MASLD/MASH, this system is relevant because mitochondrial dysfunction is not limited to reduced ATP output. Disturbances in ETC activity can alter the balance between energy production and ROS generation. When electron flow, cytochrome c transfer, cytochrome c oxidase activity, or ATP synthase coupling becomes impaired, hepatocytes may experience both bioenergetic stress and oxidative injury. This provides a mechanistic link between lipid overload, mitochondrial respiratory stress, ROS production, and downstream inflammatory or fibrotic responses.

### 2.4. Redox Homeostasis, Antioxidant Defense, and CYP2E1-Related Stress

Redox homeostasis is a central part of hepatocyte mitochondrial function. During fatty acid β-oxidation and TCA cycle activity, NADH and FADH_2_ provide electrons to the respiratory chain. Their reoxidation supports continued substrate oxidation, ATP production, and maintenance of the NADH/NAD^+^ balance. When electron transfer becomes inefficient, NADH reoxidation is impaired, the NADH/NAD^+^ ratio may rise, and mitochondrial redox balance becomes less stable. In this setting, oxidative metabolism and antioxidant defense become functionally linked rather than separate processes [38,41].

ROS in hepatocytes arise from several sources. The mitochondrial electron transport chain is a major source, especially when electron leak increases at complexes I and III. CYP2E1 provides another important source of ROS in the liver. CYP2E1 is mainly associated with the endoplasmic reticulum, but mitochondrial CYP2E1 has also been described. Under metabolic, alcohol-related, or xenobiotic stress, CYP2E1 can promote oxidative stress, mitochondrial dysfunction, and hepatocyte injury [42,43].

The main mitochondrial ROS species include superoxide and its downstream products. Superoxide can be generated when leaked electrons react with molecular oxygen. Manganese superoxide dismutase, also known as SOD2, converts mitochondrial superoxide into hydrogen peroxide. Hydrogen peroxide is less reactive than superoxide, but it must still be removed to prevent oxidative damage. Glutathione peroxidases and peroxiredoxins help reduce hydrogen peroxide and lipid hydroperoxides, using reducing equivalents supplied through glutathione- and thioredoxin-related systems [44,45].

The glutathione system is especially important for antioxidant buffering. Reduced glutathione removes peroxides and is converted to oxidized glutathione in the process. Therefore, the GSH/GSSG ratio provides a useful indicator of redox reserve. A falling GSH/GSSG ratio, rising GSSG, or increased lipid peroxidation suggests that ROS production is exceeding antioxidant buffering capacity. In experimental MASH-related settings, GSSG, ROS, lipid peroxidation, ATP loss, and mitochondrial membrane-potential changes often occur together, although these readouts should not be interpreted as mitochondria-specific without supporting functional evidence [41,46].

Oxidative stress can damage hepatocytes through several connected pathways. ROS can oxidize mitochondrial proteins, impair respiratory-chain components, and damage mitochondrial DNA. ROS can also attack polyunsaturated fatty acids in cellular and mitochondrial membranes, generating lipid peroxidation products such as malondialdehyde and 4-hydroxynonenal. These reactive products can modify proteins, nucleic acids, and membrane lipids, thereby linking redox stress to organelle dysfunction, inflammatory signaling, and cell injury [41,47].

CYP2E1-related stress is relevant because it can amplify this redox burden. Increased CYP2E1 activity has been associated with ROS overproduction, oxidative stress, mitochondrial dysfunction, necroinflammation, and fibrosis in fatty liver disease models. In fast-food diet models, CYP2E1 contributed to nitroxidative stress, ER stress, inflammation, insulin resistance, and fibrosis. These findings support the view that CYP2E1 is not only a detoxification enzyme, but also a redox stress amplifier when metabolic pressure is sustained [43,48].

Thus, ROS in MASLD/MASH should be interpreted as the result of both increased production and insufficient buffering. The relevant sequence can be summarized as follows: ETC and CYP2E1-derived ROS increase; superoxide and hydrogen peroxide are generated; SOD2, GPx, peroxiredoxins, glutathione, and thioredoxin-related systems attempt to buffer this burden; and when antioxidant capacity is exceeded, oxidative injury affects mitochondrial proteins, mtDNA, membrane lipids, ER homeostasis, and inflammatory signaling. This redox imbalance helps connect lipid overload and mitochondrial respiratory stress to steatohepatitis, hepatocyte injury, and fibrotic remodeling.

### 2.5. Adaptive Mitochondrial Compensation and Compensation Failure

Mitochondrial dysfunction in MASLD/MASH should not be interpreted only as a passive loss of mitochondrial activity. In early metabolic overload, hepatocyte mitochondria may first become over-activated as part of an adaptive response. Increased fatty acid β-oxidation, higher TCA cycle flux, enhanced oxidative phosphorylation, and more active NADH/FADH_2_ reoxidation can help hepatocytes process excess substrates and maintain ATP production [41,49,50]. This response is initially protective because it limits lipid retention and supports metabolic flexibility.

This compensatory state is regulated by nutrient- and energy-sensing pathways. PPARα promotes fatty acid oxidation programs [51], whereas PGC-1α coordinates mitochondrial biogenesis and oxidative metabolism [52]. AMPK acts as an energy sensor that favors catabolic metabolism and mitochondrial adaptation [53], and SIRT1 and SIRT3 connect NAD^+^ availability to mitochondrial biogenesis, fatty acid oxidation, antioxidant defense, and protein deacetylation [54]. Together, the PPARα/PGC-1α/AMPK/SIRT signaling network helps hepatocytes adjust mitochondrial substrate handling during fasting, nutrient excess, and early steatosis.

The key issue is that compensation has a threshold. When lipid and nutrient pressure persists, increased mitochondrial flux can become maladaptive [41,49]. Continued delivery of fatty acids and reducing equivalents places pressure on ETC complexes. If electron transfer through complexes I–IV and ATP synthesis through complex V remain well coupled, increased oxidative metabolism may still support adaptation. If respiratory efficiency declines, however, the same increased substrate flux can promote electron leak, especially at complexes I and III, and increase mitochondrial ROS production [38,41,49].

This transition explains why mitochondria can appear both activated and dysfunctional in MASLD/MASH. Upregulated oxidative flux may coexist with inefficient respiration. In that setting, mitochondria are not simply damaged from the beginning; they are first recruited to handle excess substrate, and then fail when substrate load, ETC stress, and antioxidant demand exceed adaptive capacity. The result is a shift from adaptive remodeling to pathological dysfunction [41,49].

Compensation failure involves several reinforcing mechanisms. Electron leak increases ROS production, and ROS can oxidize mitochondrial proteins, damage respiratory-chain components, and impair ATP generation [38,41]. ROS can also oxidize cardiolipin, a mitochondrial inner-membrane phospholipid that supports respiratory-chain organization and membrane integrity [55]. Cardiolipin oxidation may weaken ETC function and increase mitochondrial membrane vulnerability, thereby further amplifying respiratory stress [38,55].

Mitochondrial DNA damage provides another link between metabolic stress and disease progression. ROS-mediated injury can damage mtDNA and impair the expression of mtDNA-encoded OXPHOS components [55]. Damaged mtDNA may also escape from mitochondria and act as a danger signal, activating innate immune pathways [56]. Thus, the consequence of compensation failure is not limited to reduced ATP production. It also includes danger-signal release, inflammatory activation, and amplification of hepatocyte stress.

Failure of mitophagy and mitochondrial proteostasis further sustains this loop. Under normal conditions, damaged mitochondria can be removed or repaired through mitochondrial quality-control pathways [56,57]. With persistent metabolic stress, however, impaired mitophagy, disturbed fusion–fission balance, and defective proteostasis allow damaged mitochondria to persist [57]. These organelles can continue to generate ROS, release mitochondrial danger signals, and promote inflammatory and fibrogenic signaling.

This adaptive-failure model helps explain MASLD/MASH progression. Early mitochondrial over-activation may delay lipid accumulation and support metabolic flexibility. Once the threshold is crossed, substrate overload, ETC electron leak, ROS generation, cardiolipin oxidation, mtDNA damage, and failed mitochondrial quality control form a feed-forward loop. This loop connects lipid overload to worse metabolism, hepatocyte injury, inflammatory signaling, and fibrosis.

## 3. Mitochondrial Dysfunction as a Driver of MASLD/MASH Progression

**Figure 2:**
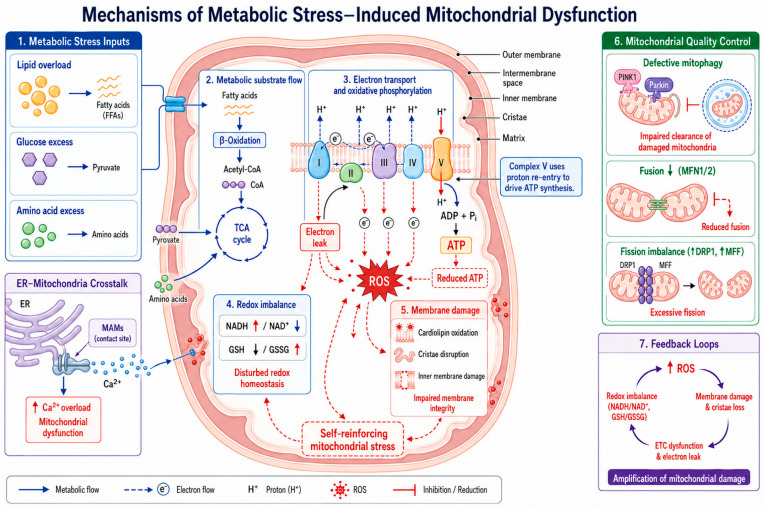
Mechanisms of metabolic stress-induced mitochondrial dysfunction.

The following section follows a practical disease sequence. Chronic metabolic pressure first tests the adaptive capacity of hepatic mitochondria. When this capacity is exceeded, the same organelles that normally clear fatty acids and maintain ATP production can become sources of redox stress, damaged mitochondrial components, and inflammatory signals. The major steps in this sequence are summarized in Figure 2.

### 3.1. Metabolic Syndrome, Insulin Resistance, and Hepatic Lipid Overload

Excess lipid input is one of the earliest events in metabolic-syndrome-related MASLD/MASH. This input does not arise from the liver alone. Obesity, type 2 diabetes, insulin resistance, hyperinsulinemia, hyperglycemia, and dyslipidemia increase the delivery and synthesis of lipids in hepatocytes [1,2]. Fatty acids reach the liver from adipose tissue lipolysis, diet, de novo lipogenesis, and triglyceride-rich lipoprotein remnants [58,59]. Hepatic lipid overload therefore reflects systemic metabolic dysfunction as well as intrahepatic lipid handling.

Adipose insulin resistance is a major upstream driver. Under physiological conditions, insulin suppresses adipose tissue lipolysis and limits the release of free fatty acids into the circulation. In obesity and type 2 diabetes, this suppression becomes incomplete. Increased adipose lipolysis then raises circulating non-esterified fatty acids, which are delivered to the liver and taken up by hepatocytes. This adipose tissue–liver fatty acid flux provides a direct link between systemic insulin resistance and hepatic steatosis [58,59].

At the same time, hyperinsulinemia and hyperglycemia stimulate hepatic de novo lipogenesis. Insulin promotes lipogenic transcription through SREBP-1c, whereas glucose and carbohydrate-derived metabolites can activate ChREBP-dependent glycolytic and lipogenic programs [23,60]. This creates a metabolically unfavorable state in which insulin fails to suppress adipose lipolysis effectively, but hepatic lipogenic signaling remains sufficiently active to promote fatty acid and triglyceride synthesis.

The fate of these lipids depends on the balance among oxidation, storage, secretion, and conversion into toxic lipid species. Triglyceride storage in lipid droplets can initially buffer excess fatty acids and may reduce acute lipotoxic stress. VLDL secretion also provides a route for triglyceride export. However, lipid droplet storage and VLDL export have limited capacity. When lipid input and de novo lipogenesis continue to exceed mitochondrial oxidation and export capacity, hepatocytes progressively accumulate triglycerides and non-triglyceride lipid intermediates [61].

The harmful effect of lipid overload depends on the lipid species that accumulate. Saturated fatty acids such as palmitate are widely used to model hepatocyte lipotoxicity [5,62] because they impair organelle function and increase cellular stress. Non-triglyceride intermediates, especially ceramides and diacylglycerols, can disturb insulin signaling, membrane properties, ER homeostasis, and mitochondrial function [63,64]. Lysophosphatidylcholine may also contribute, but current MASLD/MASH evidence is stronger for ceramides and diacylglycerols.

This distinction is useful, but it should not be read as a strict hierarchy of lipid toxicity. Triglyceride storage may be partly protective in some contexts, whereas ceramides, diacylglycerols, saturated fatty acids, and other lipid species are more closely linked to insulin resistance and organelle stress. The relative importance of each species is likely to vary with model, diet, disease stage, and cell type [63,65,66].

Mitochondria are both targets and regulators of this lipotoxic process. When mitochondrial fatty acid oxidation is sufficient, part of the lipid burden can be disposed of safely. When mitochondrial handling becomes inefficient, hepatocytes are less able to oxidize incoming fatty acids. Unused fatty acids are then more likely to remain in the cytosol, expand lipid droplets, require VLDL export, or enter pathways that generate lipotoxic intermediates.

Thus, metabolic syndrome connects to mitochondrial dysfunction through a lipid-overload sequence: adipose insulin resistance increases FFA influx; hyperinsulinemia and hyperglycemia drive SREBP-1c- and ChREBP-mediated de novo lipogenesis; lipid droplets and VLDL export initially buffer excess triglyceride; and persistent overload favors palmitate-, ceramide-, and diacylglycerol-related lipotoxicity. This upstream metabolic pressure tests mitochondrial fatty acid disposal capacity and sets the stage for oxidative stress, ER–mitochondria stress, inflammatory signaling, and fibrosis.

The gut-liver-mitochondria axis provides an additional layer linking systemic metabolism to hepatic mitochondrial stress. Gut-derived metabolites, bile acids, short-chain fatty acids, ethanol-like metabolites, endotoxin-related signals, and microbiota-driven inflammatory mediators can influence hepatic substrate flux, ER stress, redox balance, and mitochondrial function [67,68]. Conversely, impaired hepatic mitochondrial metabolism can alter bile acid signaling, lipid handling, and inflammatory tone, which may further reshape gut-liver communication. This axis should therefore be interpreted as a bidirectional metabolic and inflammatory network rather than as a single linear pathway.

### 3.2. Bioenergetic Remodeling and Loss of Metabolic Flexibility

Bioenergetic remodeling refers to changes in the way hepatocyte mitochondria generate and allocate energy during MASLD/MASH progression. It is closely related to metabolic flexibility, which is the ability of hepatocytes to switch among fuels according to nutritional state. In the normal liver, fasting favors fatty acid β-oxidation, ketogenesis, gluconeogenic support, and amino acid-derived anaplerosis, whereas feeding favors glucose utilization, glycogen handling, and lipid synthesis. In MASLD/MASH, this adaptive switching is gradually lost. The central issue is therefore not simply that mitochondria are damaged, but how mitochondrial energy metabolism moves from adaptation to overload and then to failure [23,69].

During early steatosis or simple lipid overload, mitochondrial oxidation may increase as a compensatory response. Increased fatty acid β-oxidation, TCA cycle flux, OXPHOS activity, and NADH/FADH_2_ reoxidation can help hepatocytes process excess fatty acids and maintain ATP production. This response is not necessarily pathological at first. It may be supported by nutrient-sensing and mitochondrial regulatory pathways, including PPARα, PGC-1α, AMPK, and SIRT1/SIRT3 signaling. However, increased FAO and OXPHOS also deliver more reducing equivalents to the electron transport chain. Thus, early mitochondrial activation can protect hepatocytes from lipid accumulation, but it also increases electron pressure on respiratory-chain complexes [49,70,71,72].

The transition from compensation to dysfunction occurs when lipid input exceeds mitochondrial disposal capacity. Under persistent substrate overload, β-oxidation generates large amounts of acetyl-CoA, NADH, and FADH_2_, while the TCA cycle and ETC have finite processing capacity. This can create a metabolic bottleneck characterized by incomplete fatty acid oxidation, acylcarnitine changes, altered TCA cycle intermediates, NADH/NAD^+^ imbalance, and reduced coupling efficiency. At this stage, mitochondria are not completely inactive. Rather, they enter a high-load but low-efficiency state. Sustained electron pressure then creates conditions for electron leak and later oxidative stress [72,73,74,75].

Human and experimental studies suggest that mitochondrial respiratory impairment becomes more evident in steatohepatitis and advanced disease, although the exact timing varies by model, disease stage, and measurement method. Reported abnormalities include reduced maximal respiration, impaired OXPHOS, lower ATP production, reduced respiratory-chain complex activity, decreased mitochondrial membrane potential, reduced mitochondrial biogenesis, and incomplete fatty acid oxidation. Once these changes develop, hepatocytes cannot dispose of fatty acids efficiently, which further promotes lipid retention, lipotoxicity, and metabolic stress [74,75,76,77,78].

Loss of metabolic flexibility also involves failed substrate switching. In a healthy liver, fasting shifts metabolism toward fatty acids, β-oxidation, ketogenesis, and amino acid-derived carbon entry into the TCA cycle, gluconeogenesis, and urea-cycle-related metabolism. Feeding shifts hepatocytes toward glucose metabolism, glycogen handling, and lipid synthesis. In MASLD/MASH, insulin resistance disrupts this coordination. Hepatic gluconeogenesis may remain active, de novo lipogenesis may also remain high, and the normal coordination between glucose metabolism, fatty acid oxidation, ketogenesis, and amino acid metabolism becomes weaker. BCAA metabolism and TCA anaplerosis may also be altered. As a result, ATP production, redox balance, and lipid disposal become less responsive to nutritional state [23,69,73,79].

This stage-dependent model helps connect lipid overload to downstream mitochondrial injury. In early MASLD or simple steatosis, compensatory FAO and OXPHOS may preserve part of substrate handling. In MASH, respiration can become inefficient, coupling may decline, and mitochondria enter a ROS-prone and ATP-stressed state. In fibrosis or advanced disease, impaired respiratory capacity, reduced biogenesis, quality-control failure, and persistent metabolic inflexibility become more prominent. This bioenergetic inflexibility creates the metabolic background for respiratory-chain ROS generation, lipid peroxidation, and mitochondrial quality-control failure.

### 3.3. Respiratory-Chain ROS, Lipid Peroxidation, and Ferroptosis

The bioenergetic remodeling described above provides the metabolic setting for oxidative stress. When fatty acid oxidation and oxidative phosphorylation are increased early, more NADH and FADH_2_ enter the respiratory chain. If electron transfer remains efficient, this may support adaptation. When respiratory efficiency declines, however, electron transfer becomes less stable and electron leak increases. Leaked electrons can reduce molecular oxygen to superoxide. In this sense, ROS generation is not an isolated event in MASLD/MASH, but a direct consequence of inefficient electron transfer during mitochondrial bioenergetic failure [38,41,80].

Complex I and complex III are especially important ETC-related sites of mitochondrial ROS formation. Complex I accepts electrons from NADH and transfers them to ubiquinone. When the NADH/NAD^+^ ratio is high, electron flow is restricted, or mitochondrial membrane potential is excessive, complex I can favor superoxide generation. Reverse electron transport may further increase complex I-derived ROS under conditions of high substrate pressure. Complex III can also generate superoxide, particularly at the Qo site of the Q cycle. Because complex III-derived ROS may be released toward either the matrix or the intermembrane space, it provides a route through which mitochondrial redox stress can influence intermembrane-space proteins, cytosolic signaling, and inflammatory responses [37,38,80,81].

ROS should not be treated as one single entity. Superoxide (O_2_^•−^) is the initial product of mitochondrial electron leak and is generated mainly in the matrix or intermembrane space. It is reactive and relatively limited in diffusion. Hydrogen peroxide (H_2_O_2_) is formed when superoxide is converted by superoxide dismutases, including mitochondrial SOD2. H_2_O_2_ is more stable and can act as a redox signaling molecule. Lipid peroxides are formed when ROS react with membrane lipids, especially polyunsaturated fatty acids. These oxidized lipids are closely related to membrane damage, mitochondrial dysfunction, and ferroptosis. Therefore, mild ROS production may participate in adaptive signaling, whereas sustained ROS excess shifts the system toward oxidative injury [37,38,82].

Antioxidant failure explains why ROS becomes damaging. Under physiological conditions, SOD2 converts mitochondrial superoxide to H_2_O_2_, while glutathione peroxidases and peroxiredoxins remove H_2_O_2_ and lipid hydroperoxides. The glutathione system provides a major reducing reserve. Reduced glutathione is consumed during peroxide detoxification and converted to oxidized glutathione. Thus, the GSH/GSSG ratio reflects the balance between peroxide detoxification and oxidative burden. In MASLD/MASH, persistent lipid overload, respiratory-chain pressure, and inflammatory stress can exceed this buffering capacity. When SOD2, GPx/GPX4, peroxiredoxin, glutathione, thioredoxin, or NRF2-related responses are insufficient, ROS is no longer only a signaling mediator; it becomes an injury amplifier [44,82,83,84].

Lipid peroxidation is a key route by which redox stress becomes structural mitochondrial injury. ROS can oxidize polyunsaturated fatty acids in cellular and mitochondrial membranes, reducing membrane integrity and generating reactive aldehydes. Malondialdehyde and 4-hydroxynonenal are commonly used products or readouts of lipid peroxidation. 4-hydroxynonenal can modify proteins, lipids, and DNA, including mitochondrial enzymes and respiratory-chain proteins, thereby worsening bioenergetic dysfunction. Cardiolipin is particularly relevant because it is enriched in the inner mitochondrial membrane and supports cristae architecture, ETC organization, and cytochrome c interaction. Cardiolipin oxidation or abnormal remodeling can reduce respiratory-chain efficiency, increase membrane vulnerability, and may facilitate cytochrome c mobilization. Thus, lipid peroxidation is not only a marker of oxidative stress, but also a mechanism that further impairs mitochondrial function [83,85,86,87,88].

Ferroptosis provides an additional link between lipid peroxidation and hepatocyte injury. Ferroptosis is an iron-dependent, lipid peroxide-driven, non-apoptotic form of regulated cell death. Its key features include iron dysregulation, accumulation of lipid peroxides, ACSL4-related incorporation of polyunsaturated fatty acids into phospholipids, GPX4 insufficiency, glutathione depletion, and impaired cystine import through system Xc^−^/SLC7A11. Mitochondria are not the sole executors of ferroptosis in all models. However, in MASLD/MASH, mitochondrial ROS, cardiolipin vulnerability, redox imbalance, and lipid overload can create conditions that favor ferroptotic injury. Experimental and animal studies support a role for ferroptosis-related pathways in steatohepatitis, lipid peroxidation, inflammation, and fibrosis, whereas human MASLD/MASH evidence is still more often based on markers, associations, or specific high-risk settings rather than direct longitudinal proof. Ferroptosis should therefore be presented as an emerging and plausible injury mechanism, not as the sole explanation for hepatocyte death in human MASH [89,90,91,92,93].

Together, respiratory-chain inefficiency, antioxidant-buffering failure, lipid peroxidation, and ferroptosis form a feed-forward injury loop. Inefficient electron transfer increases superoxide and H_2_O_2_ production; insufficient antioxidant defense allows ROS and lipid peroxides to accumulate; lipid peroxidation damages membranes, cardiolipin, respiratory proteins, and mitochondrial DNA; and ferroptosis-related hepatocyte injury can amplify inflammation and fibrosis. This sequence connects metabolic overload and bioenergetic failure to the mitochondrial quality-control and danger-signal pathways discussed below.

### 3.4. ER–Mitochondria, Lysosome–Mitochondria, and Peroxisome–Mitochondria Crosstalk

Mitochondrial dysfunction in MASLD/MASH should be viewed as part of a broader organelle stress network. Hepatocyte lipid metabolism depends on coordinated communication among mitochondria, the endoplasmic reticulum (ER), lysosomes, lipid droplets, and peroxisomes. Mitochondria support fatty acid β-oxidation, TCA cycle activity, oxidative phosphorylation, ATP production, and redox balance. The ER supports lipid synthesis, protein folding, Ca^2+^ storage, and unfolded protein response signaling. Lysosomes regulate autophagy, mitophagy, and lipid droplet turnover, whereas peroxisomes participate in very-long-chain fatty acid oxidation, plasmalogen and bile-acid-related metabolism, and ROS handling. In MASLD/MASH, these organelles are not injured in isolation. Organelle crosstalk explains why lipid overload can simultaneously disturb metabolism, redox balance, proteostasis, calcium signaling, and mitochondrial quality control [94,95,96,97].

ER–mitochondria contact sites, often referred to as mitochondria-associated membranes (MAMs), are central to this network. MAMs are specialized contact regions between the ER and mitochondria that regulate Ca^2+^ transfer, lipid exchange, mitochondrial dynamics, autophagy-related signaling, and metabolic adaptation. In hepatocytes, altered MAM integrity can affect mitochondrial oxidative capacity, triglyceride handling, cholesterol metabolism, and lipid droplet behavior. In MASLD/MASH, lipid overload may remodel ER–mitochondria contacts. This remodeling should not be interpreted as uniformly harmful or uniformly protective. MAM changes may support early adaptation by improving metabolic coupling, but they can become maladaptive when excessive Ca^2+^ transfer, lipid imbalance, and ER stress impair mitochondrial respiration and quality control [94,95,96,97,98].

Ca^2+^ transfer provides a direct mechanism linking ER stress to mitochondrial dysfunction. The ER is a major intracellular Ca^2+^ reservoir, and Ca^2+^ can be transferred to mitochondria through MAM-associated channels and tethering complexes. Physiological Ca^2+^ transfer supports metabolic coupling because mitochondrial Ca^2+^ can activate TCA cycle dehydrogenases and enhance ATP production. However, excessive or poorly regulated Ca^2+^ transfer can cause mitochondrial Ca^2+^ overload, disturb membrane potential, increase ROS generation, increase the propensity for mitochondrial permeability transition, and impair respiration. In MASLD/MASH, lipotoxic ER stress and altered ER–mitochondria contacts may therefore convert Ca^2+^ signaling from a metabolic coupling mechanism into a mitochondrial stress amplifier [65,95,97].

The unfolded protein response (UPR) is another key component of ER–mitochondria crosstalk. PERK activation reduces global protein translation through eIF2α and can induce ATF4 and CHOP-dependent stress responses. When prolonged, PERK–ATF4–CHOP signaling may promote oxidative stress, apoptosis-prone signaling, and mitochondrial dysfunction. IRE1α activates XBP1 splicing and participates in protein folding, lipid metabolism, and inflammatory signaling; chronic IRE1α activation can also engage JNK and inflammatory pathways. ATF6 translocates to the Golgi and induces ER chaperones that support ER proteostasis. Thus, UPR signaling is initially protective, but chronic activation shifts hepatocytes from adaptive proteostasis toward inflammatory and pro-death signaling. This transition helps explain how persistent lipid overload links ER stress to mitochondrial injury and MASH progression [65,99,100,101].

Lysosome–mitochondria crosstalk determines whether mitochondrial injury remains reversible. Autophagy is the broader degradative process by which cytoplasmic material, lipid droplets, and damaged organelles are delivered to lysosomes. Mitophagy is the selective removal of damaged mitochondria. Efficient mitophagy requires autophagosome formation, autophagosome–lysosome fusion, lysosomal acidification, and degradation of mitochondrial cargo. In MASLD/MASH, lipid overload can impair lysosomal acidification and autophagic flux, leading to LC3-II and p62/SQSTM1 accumulation, defective lipophagy, and insufficient mitochondrial clearance. When damaged mitochondria are not removed, they continue to generate ROS and become potential sources of mitochondrial danger signals. In this sense, lysosomal dysfunction converts mitochondrial damage from a reversible quality-control problem into persistent organelle stress [102,103,104,105,106].

Peroxisome–mitochondria crosstalk adds another layer to hepatic lipid handling. Peroxisomes oxidize very-long-chain fatty acids and branched-chain fatty acids, shorten fatty acyl chains, and generate products that can be further metabolized by mitochondria. Peroxisomes also participate in plasmalogen metabolism, bile-acid-related pathways, and ROS handling. Unlike mitochondrial β-oxidation, peroxisomal β-oxidation generates H_2_O_2_, which is normally buffered by catalase and related antioxidant systems. During metabolic overload, demand increases on both peroxisomal and mitochondrial oxidation. If peroxisomal FAO, catalase activity, or peroxisome–mitochondria substrate transfer becomes insufficient, lipid retention and redox imbalance may worsen. Peroxisomes should therefore not be viewed as a duplicate of mitochondria, but as complementary organelles that help determine whether fatty acid overload is safely processed or converted into oxidative and lipotoxic stress [107,108,109,110].

Together, ER, lysosomal, and peroxisomal stress form a feed-forward organelle network. ER stress can promote Ca^2+^ overload, lipid imbalance, and UPR-dependent inflammatory signaling, thereby impairing mitochondrial function. Lysosomal dysfunction can block autophagic flux and mitophagy, allowing damaged mitochondria and lipid droplets to accumulate. Peroxisomal dysfunction can alter very-long-chain fatty acid handling and increase H_2_O_2_-related redox stress. These processes reinforce mitochondrial respiratory stress, ROS production, and metabolic inflexibility. This organelle network provides the background for impaired mitochondrial quality control, mtDNA instability, and mitochondrial danger-signal release in later stages of MASLD/MASH.

### 3.5. Mitochondrial Dynamics, Mitophagy, Proteostasis, and mtDNA Instability

Hepatocyte mitochondria are exposed to sustained lipid overload, ROS, ER stress, Ca^2+^ stress, and inflammatory signals during MASLD/MASH progression. To maintain function under these conditions, mitochondria depend on a coordinated quality-control network that includes fission, fusion, mitophagy, proteostasis, and mtDNA maintenance [38,111,112,113]. These processes are not independent. Mitochondrial dynamics reshape the network, mitophagy removes damaged organelles, proteostasis maintains the quality of imported and respiratory-chain proteins, and mtDNA stability supports the expression of mtDNA-encoded OXPHOS subunits. The major problem in MASH is therefore not one isolated protein change, but failure of an integrated mitochondrial quality-control system.

Mitochondrial fission divides the mitochondrial network into smaller units. This process supports mitochondrial distribution, metabolic adaptation, and the segregation of damaged mitochondria before mitophagy. DRP1 is the major fission GTPase and is recruited to the outer mitochondrial membrane through receptors and regulatory proteins such as FIS1 and MFF [114,115]. Phosphorylated DRP1 is often used as a readout of fission activity, although different phosphorylation sites can have different functional meanings. In MASLD/MASH, fission must be interpreted carefully. Moderate fission can be protective when it isolates damaged mitochondria for clearance, whereas excessive fission can promote fragmentation, membrane-potential loss, ROS generation, and respiratory impairment [101,114,116,117]. Conversely, insufficient fission can also be harmful because damaged mitochondria may not be efficiently separated for removal.

DRP1 activity is also regulated by phosphorylation, and phosphorylated DRP1 (p-DRP1) is often used as a marker of mitochondrial fission [114,115]. However, p-DRP1 should be interpreted carefully because different phosphorylation sites may have different functional effects. Increased DRP1 phosphorylation at pro-fission sites can indicate mitochondrial fragmentation, whereas other phosphorylation events may inhibit DRP1 recruitment or activity. Thus, p-DRP1 is useful as a mechanistic readout only when the phosphorylation site and mitochondrial morphology are specified.

Mitochondrial fusion complements fission by preserving network integrity. Fusion allows mitochondrial content mixing, helps maintain membrane potential, supports mtDNA stability, and buffers local mitochondrial damage. MFN1 and MFN2 mediate outer mitochondrial membrane fusion, whereas OPA1 regulates inner mitochondrial membrane fusion and cristae architecture [112,118,119]. MFN2 also participates in ER–mitochondria contacts [95,118], linking mitochondrial fusion to MAM organization and lipid/Ca^2+^ communication. In MASLD/MASH, impaired fusion can contribute to mitochondrial fragmentation, reduced respiratory efficiency, mtDNA instability, and ROS-prone metabolism. OPA1 disruption may affect cristae structure and OXPHOS organization, while MFN2 loss may impair both fusion and ER–mitochondria crosstalk. Thus, fusion maintains mitochondrial network resilience; when fusion is impaired, hepatocytes lose the ability to buffer local mitochondrial injury.

Mitophagy is the selective removal of damaged mitochondria. It is especially important for clearing depolarized, ROS-producing, or mtDNA-damaged mitochondria before they become persistent sources of stress signals. In the PINK1/Parkin pathway, loss of mitochondrial membrane potential allows PINK1 to accumulate on the outer mitochondrial membrane. PINK1 then promotes Parkin recruitment and activation [103,104], leading to ubiquitination of outer mitochondrial membrane proteins and recruitment of autophagy machinery. Receptor-mediated pathways also contribute. BNIP3, NIX, and FUNDC1 can promote PINK1/Parkin-independent mitophagy under hypoxia [120,121,122], metabolic stress, mitochondrial depolarization, or fibrotic injury. In MASH, impaired mitophagy allows damaged mitochondria to persist, sustaining ROS production, mtDNA injury, respiratory dysfunction, and inflammatory signaling.

Mitophagy depends on the broader autophagy machinery. LC3-II reflects autophagosome formation, p62/SQSTM1 can act as a cargo adaptor and may also accumulate when autophagic flux is blocked, and ATG5/ATG7 are required for autophagosome formation. These markers should not be interpreted as static proof of increased or decreased autophagy. LC3-II and p62 changes need to be evaluated together with lysosomal flux, because their accumulation may reflect either enhanced autophagosome formation or impaired degradation. In MASLD/MASH, lipid overload and lysosomal dysfunction can impair autophagic flux, leading to defective lipophagy and defective mitophagy at the same time. This failure increases both lipid droplet accumulation and damaged mitochondrial accumulation [121,123,124,125].

Beclin-1 should also be considered when interpreting autophagy-related mitochondrial quality control. Beclin-1 participates in autophagosome nucleation and helps initiate autophagic cargo processing [126], but its expression alone does not prove effective mitophagy. Like LC3-II and p62, Beclin-1 should be interpreted together with lysosomal flux, ATG5/ATG7-dependent autophagosome formation, and mitochondrial cargo degradation.

Mitochondrial proteostasis provides another layer of quality control. Mitochondrial proteins are encoded by both nuclear and mitochondrial genomes, and OXPHOS complexes require coordinated protein import, folding, assembly, repair, and degradation. mtHSP70/HSPA9 participates in mitochondrial protein import and folding, HSP60 supports matrix protein folding, and mitochondrial proteases such as LONP1 and ClpP remove misfolded, oxidized, or damaged proteins [123,124,125]. The mitochondrial unfolded protein response is an adaptive transcriptional response that attempts to restore mitochondrial protein homeostasis under proteotoxic stress [124]. In MASLD/MASH, ROS and lipid aldehydes can modify respiratory-chain proteins and impair ETC assembly [127,128,129,130]. When proteostasis fails, damaged proteins accumulate, OXPHOS efficiency declines, ROS generation increases, and mitochondria become more dependent on mitophagy. If proteostasis and mitophagy fail together, damaged mitochondria persist as long-term metabolic and inflammatory stress sources.

The mitochondrial unfolded protein response (UPRmt) is an adaptive stress response that attempts to restore mitochondrial proteostasis when misfolded or damaged mitochondrial proteins accumulate [124]. UPRmt-related markers, including HSP60, HSP90, mtHSP70/HSPA9, LONP1, and ClpP, may indicate mitochondrial proteotoxic stress or attempted recovery. However, persistent UPRmt activation may also reflect unresolved mitochondrial damage. Therefore, UPRmt should be framed as a context-dependent adaptive response rather than as uniformly protective.

mtDNA instability links mitochondrial quality control to both bioenergetic failure and innate immune activation. mtDNA copy number can reflect mitochondrial content or biogenesis, but it should not be used alone as a direct measure of mitochondrial function because it may rise during compensation or fall during advanced injury [130]. mtDNA is located close to the respiratory chain and is vulnerable to oxidative damage. Oxidative mtDNA lesions, strand breaks, deletions, or mutations can impair mtDNA-encoded OXPHOS subunits, thereby worsening ETC function [127,128]. When damaged mitochondria are not repaired or removed, mtDNA can reach the cytosol or extracellular space. Cytosolic mtDNA may activate cGAS–STING, AIM2, or NLRP3-related pathways, while circulating mtDNA may have biomarker potential. However, its cellular origin, disease-stage specificity, and clinical interpretation still require caution [127,128,129,130,131].

Mitochondrial biogenesis and mtDNA maintenance provide another layer of quality control. PGC-1alpha can coordinate mitochondrial biogenesis programs, whereas NRF1 regulates nuclear-encoded mitochondrial genes and TFAM supports mtDNA packaging, transcription, replication, and copy-number maintenance [132,133]. In MASLD/MASH, changes in NRF1 or TFAM may reflect altered mitochondrial biogenesis or mtDNA maintenance, but they do not necessarily indicate improved mitochondrial function. These markers should be interpreted together with respiratory capacity, mtDNA integrity, OXPHOS protein expression, and disease stage.

Overall, mitochondrial quality-control failure creates a feed-forward mechanism in MASLD/MASH. Fission–fusion imbalance destabilizes the mitochondrial network; mitophagy failure allows damaged mitochondria to accumulate; autophagic flux impairment prevents proper cargo degradation; proteostasis failure compromises respiratory-chain assembly; and mtDNA instability links respiratory dysfunction to inflammatory signaling. Once damaged mitochondria are not removed or repaired, they become persistent sources of ROS, mtDNA, mitochondrial RNA, and other injury signals that can activate non-parenchymal liver cells and promote progression toward inflammation and fibrosis.

### 3.6. Mitochondrial Danger Signals, Extracellular Vesicles, and Multicellular Inflammation

MASLD/MASH begins largely as hepatocyte-centered metabolic stress, but disease progression requires a multicellular inflammatory response. Lipid overload, respiratory-chain stress, ROS production, mitophagy failure, and mtDNA injury can convert damaged hepatocyte mitochondria into sources of inflammatory signals. These signals include mitochondrial DAMPs such as mtDNA, mitochondrial RNA, cardiolipin, extracellular ATP, HMGB1, oxidized lipids, and extracellular vesicle-associated mitochondrial cargo. Because mitochondria retain bacterial-like molecular features, mitochondrial nucleic acids and membrane lipids can be recognized by innate immune pathways. In this way, hepatocyte mitochondrial injury is translated into sterile inflammation in the liver [127,129,134,135].

mtDNA is one of the best-characterized mitochondrial danger signals. Cytosolic mtDNA can be sensed by cGAS, leading to cGAMP formation, STING activation, and downstream TBK1/IRF3 and NF-κB signaling. This pathway can promote type I interferon responses and inflammatory cytokine production. mtDNA can also contribute to AIM2 inflammasome activation because AIM2 recognizes cytosolic double-stranded DNA, leading to caspase-1 activation and IL-1β/IL-18 maturation. In addition, mitochondrial ROS, mtDNA leakage, cardiolipin exposure, ion fluxes, and metabolic stress can promote NLRP3 inflammasome activation, especially in Kupffer cells and recruited macrophages. These pathways should not be interpreted as universally activated in every setting. Rather, mtDNA may activate different DNA-sensing and inflammasome pathways depending on cell type, disease stage, mitochondrial damage severity, and subcellular localization [129,134,135,136,137].

Mitochondrial RNA adds another layer to this danger-signal model. Damaged mitochondria may release mitochondrial RNA, including mitochondrial double-stranded RNA, into the cytosol or extracellular space. These RNA species can engage RNA-sensing pathways such as TLR3, MDA5, and related downstream IRF3/NF-κB signaling. Compared with mtDNA, mtRNA-related mechanisms are less established in MASLD/MASH and should be presented as an emerging area. Nevertheless, available experimental evidence suggests that mitochondria-driven innate immunity in metabolic liver disease is not restricted to DNA sensing, but can include several nucleic acid-sensing routes that converge on inflammatory cytokine production [138,139].

Other DAMPs can reinforce this response. Cardiolipin normally resides in the inner mitochondrial membrane, but mitochondrial injury can promote cardiolipin oxidation or abnormal exposure, linking membrane damage to inflammasome-related signaling and mitochondrial vulnerability. Extracellular ATP released from stressed or dying cells can activate purinergic receptors such as P2X7 receptor and promote inflammasome activation. HMGB1, although nuclear rather than mitochondrial in origin, can be released by injured hepatocytes and activate TLR4- or RAGE-related inflammatory pathways. These signals do not act in isolation. In MASH, hepatocyte injury likely produces a mixed inflammatory signal composed of mitochondrial nucleic acids, oxidized lipids, ATP, HMGB1, cytokines, chemokines, and vesicle-associated cargo [135,140,141,142].

Extracellular vesicles provide a mechanism by which hepatocyte stress signals are transferred to other liver cells. Injured or lipotoxic hepatocytes can release EVs carrying lipids, proteins, coding and non-coding RNAs, mitochondrial DNA, oxidized lipid species, iron-related cargo, and other DAMP-like molecules. These vesicles can be taken up by Kupffer cells, recruited macrophages, hepatic stellate cells, endothelial cells, and other recipient cells. In experimental NASH-related settings, hepatocyte-derived EVs can promote macrophage chemotaxis, inflammatory recruitment, stellate cell migration, myofibroblast-like changes, and fibrogenic remodeling. EV-mediated mitochondrial and lipotoxic signaling is therefore an important hepatocyte-to-immune-cell and hepatocyte-to-HSC communication route, although its longitudinal human validation remains less mature than the experimental evidence [143,144,145,146,147].

The inflammatory response that follows mitochondrial danger signaling is multicellular. Kupffer cells and recruited macrophages sense DAMPs, lipotoxic mediators, and EV cargo, activate NF-κB, STING, and inflammasome pathways, and secrete TNF-α, IL-1β, IL-6, CCL2, and other inflammatory mediators. These signals recruit additional inflammatory monocytes and amplify hepatocyte injury [148]. Neutrophils can contribute through ROS release, proteases, and NET-associated injury. T cells, NK cells, NKT cells, dendritic cells, and B cells can also shape the inflammatory microenvironment through cytokine production, immune-cell recruitment, and hepatocyte killing [149]. Thus, mitochondrial DAMPs and EV-mediated signals do not simply mark hepatocyte damage; they help reshape the hepatic microenvironment. These inflammatory signals create the setting in which hepatic stellate cells become activated and extracellular matrix deposition begins [150].

### 3.7. Stellate Cell Activation, Fibrosis Progression, and Fibrosis Reversibility

Within this inflammatory microenvironment, hepatic stellate cells become the main effector cells that convert mitochondrial and immune stress into fibrotic matrix deposition. In healthy liver, hepatic stellate cells are quiescent, vitamin A-storing cells. Chronic hepatocyte injury, macrophage activation, oxidative stress, and inflammatory cytokines stimulate HSC activation. Activated HSCs undergo a myofibroblast-like transition, increase α-SMA expression, upregulate fibrogenic genes such as COL1A1 and TIMP1, and produce extracellular matrix. This HSC-centered fibrogenic program is a major driver of fibrosis in experimental and human liver injury [22,151,152].

Mitochondrial dysfunction promotes fibrosis less by collagen production inside hepatocytes and more by reshaping the inflammatory and fibrogenic microenvironment. Hepatocyte-derived DAMPs, EVs, oxidized lipids, cytokines, and chemokines can act directly on HSCs or indirectly through Kupffer cells and recruited macrophages. Macrophage-derived TNF-α, IL-1β, IL-6, TGF-β, PDGF, CCL2, and related mediators further promote HSC activation and survival. This creates a feed-forward loop: mitochondrial injury promotes inflammatory signaling, inflammatory cells amplify hepatocyte stress, and HSCs integrate these signals into extracellular matrix production [22,153,154,155].

TGF-β is a central profibrotic pathway in this process. TGF-β/SMAD signaling promotes myofibroblast differentiation, α-SMA expression, collagen synthesis, and ECM accumulation. Mitochondrial ROS, lipid peroxidation, ER stress, and macrophage-derived inflammatory mediators can strengthen TGF-β-rich signaling environments. Damaged hepatocytes and activated macrophages therefore contribute to fibrosis not only through direct cell death or cytokine release, but also by sustaining a microenvironment in which TGF-β signaling becomes dominant. This connects inflammation to ECM deposition and explains why mitochondrial stress can indirectly drive fibrogenesis [22,151,156].

Several more specific hepatocyte-to-HSC mechanisms have also been described. Osteopontin is an inflammatory and fibrogenic mediator that can promote HSC activation, migration, and ECM production. In NASH-related models, hepatocyte Notch activation can induce Sox9-dependent osteopontin production and thereby activate stellate cells. CHCHD2 provides a related example: increased hepatocyte CHCHD2 can promote liver fibrosis through Notch signaling and osteopontin upregulation. Vesicle-mediated transfer provides another route. Iron-containing hepatocyte-derived EVs can redistribute iron toward HSCs, increase HSC ROS production, and promote fibrogenic activation. These examples show that hepatocyte mitochondrial and stress-related signals can promote fibrosis through soluble mediators, developmental signaling pathways, and vesicle-mediated cargo transfer [157,158,159,160].

Fibrosis progression is an active remodeling process rather than passive scar formation. Activated HSCs and other myofibroblast-like cells produce collagen I, collagen III, fibronectin, laminin, and other matrix components. TIMPs reduce matrix degradation, while ECM stiffness feeds back to maintain HSC activation. Macrophage polarization, endothelial dysfunction, ductular reaction, and immune-cell infiltration further shape this fibrogenic niche. When mitochondrial injury and inflammation persist, ECM deposition, tissue stiffness, HSC activation, and inflammatory signaling reinforce one another. This explains why fibrosis can continue even after the initial metabolic insult is partly reduced [22,137,150,151,161].

Fibrosis should not be presented as a completely irreversible endpoint. If metabolic stress, lipotoxicity, inflammation, and profibrotic signaling decline, fibrosis can partially regress. Potential mechanisms include activated HSC apoptosis, HSC inactivation, senescence or immune-mediated clearance of activated fibrogenic cells, matrix metalloproteinase-mediated ECM degradation, macrophage phenotype switching toward restorative programs, reduced TGF-β signaling, and improved mitochondrial function, mitophagy, and redox balance. However, the reversibility of advanced cirrhosis is limited, and the degree to which mitochondrial recovery directly drives fibrosis regression in human MASH remains incompletely defined. Thus, fibrosis regression is biologically plausible and increasingly supported by experimental and therapeutic evidence, but it should be discussed as stage-dependent and context-dependent rather than guaranteed [162,163].

**Figure 3:**
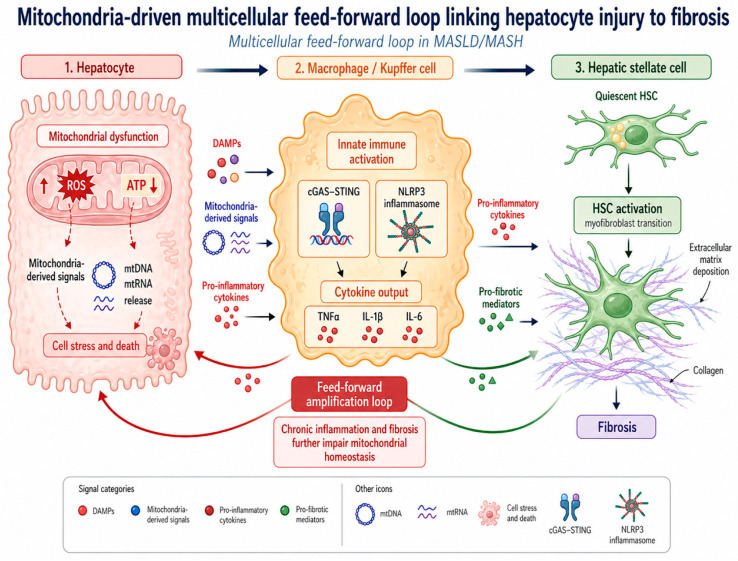
Mitochondria-driven multicellular feed-forward loop linking hepatocyte injury to fibrosis.

Together, Section 3.6 and Section 3.7 define the multicellular phase of mitochondrial dysfunction in MASLD/MASH, as summarized in Figure 3. Damaged mitochondria release danger signals and vesicle-associated cargo; innate immune cells convert these signals into inflammatory cytokines and chemokines; stellate cells respond by adopting a fibrogenic myofibroblast-like phenotype; and ECM remodeling determines whether fibrosis progresses or regresses. This sequence links hepatocyte mitochondrial damage to tissue-level inflammation, fibrosis, and potential disease resolution.

### 3.8. Stage-Dependent Mitochondrial Remodeling in MASLD/MASH

The mitochondrial changes described above should be interpreted in a stage-dependent manner. MASLD/MASH does not follow a single linear pattern in which mitochondrial function simply declines from the beginning. In early MASLD, hepatocyte mitochondria may retain compensatory capacity and may even show preserved or increased oxidative activity in some models. During MASH, the same oxidative response can become inefficient, ROS-prone, and coupled to organelle stress. During fibrosis, mitochondrial dysfunction extends beyond hepatocyte metabolism and becomes a tissue-level amplifier through DAMP release, extracellular vesicle transfer, immune-cell activation, and stellate-cell stimulation. In cirrhosis or advanced disease, mitochondrial injury is more likely to involve impaired respiratory capacity, reduced bioenergetic reserve, mtDNA instability, defective quality control, persistent inflammation, and reduced regenerative capacity. This framework helps reconcile apparently conflicting findings in the literature, because increased FAO/OXPHOS and reduced mitochondrial respiration may represent different disease stages, models, cell populations, or measurement conditions [49,164,165,166].

In early MASLD or simple steatosis, lipid input increases through adipose insulin resistance, dietary lipid handling, and de novo lipogenesis. Hepatocytes initially attempt to buffer this substrate load through lipid droplet storage, VLDL export, and mitochondrial oxidation. In this stage, mitochondrial fatty acid β-oxidation, TCA cycle activity, OXPHOS, and mitochondrial biogenesis may be preserved or adaptively increased in some settings. This response can help limit lipid retention and maintain metabolic flexibility. However, it also increases delivery of NADH and FADH_2_ to the respiratory chain. Thus, early mitochondrial adaptation is not necessarily pathological, but it creates a vulnerable state in which persistent substrate pressure can increase electron pressure and oxidative risk [49,164,167].

The transition to MASH reflects a shift from compensation to inefficient stress handling. Fatty acid oxidation may remain active, but it becomes less well coupled to ATP production and less able to dispose of lipid safely. Respiratory-chain pressure, electron leak, ROS generation, lipid peroxidation, ER stress, Ca^2+^ dysregulation, MAM remodeling, antioxidant-buffering failure, and impaired autophagy/mitophagy begin to interact. In this stage, the central problem is no longer steatosis alone, but the combination of lipid overload, mitochondrial inefficiency, redox imbalance, organelle crosstalk failure, and inflammatory injury. Ferroptosis-related lipid-peroxide injury may contribute in experimental settings, but it should be considered an emerging and context-dependent mechanism rather than the dominant explanation for all human MASH [38,75,104,165].

In fibrosis, mitochondrial dysfunction becomes a multicellular signal rather than only a hepatocyte metabolic defect. Damaged hepatocyte mitochondria can release or expose mtDNA, mtRNA, cardiolipin, ATP, HMGB1, oxidized lipids, and extracellular vesicle-associated cargo. These signals activate Kupffer cells, recruited macrophages, neutrophils, and other immune cells through pathways such as cGAS–STING, AIM2, NLRP3, TLR-related signaling, and NF-κB-dependent cytokine production. The resulting inflammatory microenvironment activates hepatic stellate cells through TGF-β, PDGF, CCL2, osteopontin, Notch-related signaling, iron-containing EVs, and other fibrogenic mediators. At this stage, mitochondrial dysfunction promotes fibrosis less by producing collagen within hepatocytes and more by reshaping the liver microenvironment into one that favors HSC activation and extracellular matrix deposition [22,135,137,151].

In cirrhosis or advanced disease, mitochondrial dysfunction becomes less readily reversible. Hepatocytes may show reduced bioenergetic reserve, impaired OXPHOS, lower ATP production, reduced mitochondrial biogenesis, mtDNA copy-number changes or mtDNA instability, persistent oxidative stress, severe quality-control failure, and impaired regenerative capacity. Non-parenchymal cells are also metabolically remodeled, including macrophages, activated stellate cells, endothelial cells, and immune-cell populations. Nevertheless, fibrosis should not be described as an absolutely irreversible endpoint. When metabolic injury, lipotoxicity, inflammatory signaling, and profibrotic pressure decline, fibrosis may partially regress through HSC apoptosis or inactivation, matrix degradation, macrophage phenotype switching, and improved mitochondrial redox and quality-control function. This potential is stage-dependent, and advanced cirrhosis has more limited reversibility than earlier fibrotic disease [22,113,166].

## 4. Translational Readouts and Therapeutic Perspectives

### 4.1. Clinical and Translational Readouts of Mitochondrial Dysfunction

Mitochondrial dysfunction is mechanistically important in MASLD/MASH, but it remains difficult to assess directly in routine clinical practice. Hepatic mitochondrial flux, substrate oxidation, respiratory coupling, and ATP production are not easily measured in patients. Liver biopsy can provide tissue-level information, but it is invasive, usually cross-sectional, affected by sampling variability, and not suitable for repeated monitoring in most clinical settings. Blood-based metabolomics, lipidomics, redox markers, and circulating mtDNA are more feasible for clinical translation, but they lack sufficient specificity for hepatocyte mitochondria. Therefore, these measurements should be interpreted as supportive readouts of mitochondrial stress rather than standalone diagnostic biomarkers of mitochondrial dysfunction [74,75,168,169].

Human liver biopsy studies remain the most direct source of evidence for hepatic mitochondrial remodeling. Biopsy samples can be used to assess mitochondrial morphology, ultrastructural abnormalities, respiratory-chain protein expression, mtDNA copy number, mtDNA mutations or deletions, oxidative injury, and mitochondrial gene-expression profiles. When fresh tissue is available, respirometry or substrate oxidation assays can provide functional information about FAO, OXPHOS, coupling efficiency, and respiratory capacity. Such studies are valuable because they evaluate mitochondria in the diseased liver itself. However, biopsy-based evidence is not easily scalable: fresh tissue is difficult to obtain, sample size is often limited, tissue composition is heterogeneous, and longitudinal follow-up is uncommon. Thus, biopsy-based studies are important for mechanistic validation, but they are not yet practical tools for routine mitochondrial assessment [74,75,170,171].

Metabolomics can provide pathway-level information about mitochondrial substrate handling. Acylcarnitines are commonly used to infer fatty acid transport, β-oxidation pressure, or incomplete fatty acid oxidation. Accumulation of long-chain or medium-chain acylcarnitines may suggest that fatty acid input exceeds mitochondrial disposal capacity, but this finding cannot by itself distinguish increased FAO, impaired FAO, or incomplete oxidation. TCA cycle intermediates, including citrate, succinate, fumarate, malate, and α-ketoglutarate, may indicate remodeling of central carbon metabolism, anaplerosis, or altered mitochondrial flux. However, these metabolites are strongly influenced by fasting status, diet, exercise, insulin resistance, diabetes, amino acid metabolism, medication exposure, and sample handling. Therefore, acylcarnitines and TCA intermediates should be interpreted as metabolic clues rather than direct measurements of mitochondrial function [16,70,172,173,174,175].

Redox-related readouts add another translational layer. The lactate/pyruvate ratio may reflect cytosolic redox state, because impaired oxidative metabolism can favor conversion of pyruvate to lactate. However, this ratio is not liver-specific and may be affected by systemic glucose metabolism, hypoxia, exercise, sepsis, medications, and sampling conditions. The glutathione system is also informative. Reduced glutathione is consumed during peroxide detoxification and converted to oxidized glutathione, making the GSH/GSSG ratio a useful indicator of antioxidant reserve. A lower GSH/GSSG ratio or increased GSSG may support the presence of oxidative stress in MASLD/MASH, particularly when considered together with ROS, lipid peroxidation, ATP levels, and mitochondrial membrane potential. Nevertheless, GSH/GSSG is not mitochondria-specific and cannot identify whether ROS originate from the ETC, CYP2E1, peroxisomes, NOX enzymes, inflammatory cells, or other sources [176,177,178,179].

Mitochondrial lipidomics is mechanistically attractive, especially cardiolipin-focused profiling. Cardiolipin is enriched in the inner mitochondrial membrane and supports cristae architecture, respiratory-chain organization, supercomplex stability, ATP production, and cytochrome c interaction. Cardiolipin remodeling or oxidation may therefore indicate mitochondrial membrane stress and impaired respiratory organization. Phosphatidic acid and other mitochondrial or mitochondria-associated phospholipid species may also provide information about membrane remodeling and organelle stress. However, cardiolipin and related mitochondrial lipid species are not yet routine clinical biomarkers. Their measurement usually requires tissue or high-quality biospecimens, specialized lipidomics platforms, careful normalization, and validation across disease stages and treatment settings. Cardiolipin-focused lipidomics is therefore mechanistically informative but remains primarily translational rather than routine clinical [180,181,182,183].

mtDNA-related measurements are useful but require cautious interpretation. Tissue mtDNA copy number can reflect mitochondrial content, biogenesis, or mtDNA maintenance, but it is not equivalent to mitochondrial function. mtDNA copy number may increase as a compensatory response in early metabolic stress or decline in advanced injury, and changes may differ by model, tissue, and disease stage. Blood or peripheral blood mononuclear cell mtDNA copy number is easier to measure, but it does not necessarily represent hepatocyte mtDNA biology. Circulating mtDNA may provide a readout of mitochondrial damage and DAMP release and may correlate with inflammation, cell injury, or fibrosis severity. However, circulating mtDNA is not liver-specific and may originate from hepatocytes, immune cells, platelets, endothelial cells, or sample-related hemolysis and cell lysis. Pre-analytical handling, plasma versus serum selection, DNA extraction methods, and inflammatory comorbidities can strongly affect results. Thus, circulating mtDNA is currently best viewed as an exploratory biomarker of mitochondrial injury and sterile inflammation rather than as a specific diagnostic marker of hepatic mitochondrial dysfunction [130,184,185,186].

Taken together, no single clinical test currently provides a specific, validated, and clinically mature diagnosis of hepatic mitochondrial dysfunction in MASLD/MASH. The most realistic strategy is a multimodal panel that integrates biopsy-based evidence, metabolomics, lipidomics, redox markers, mtDNA-related measurements, histology, imaging, clinical phenotype, and treatment response. Such a panel should also be interpreted through a stage-dependent framework: early MASLD may show adaptive metabolic remodeling; MASH may show inefficient respiration, oxidative stress, and organelle injury; fibrosis may show DAMP release and multicellular signaling; and advanced disease may show reduced bioenergetic reserve and impaired repair capacity. Future biomarker studies should prioritize longitudinal human cohorts and validate sensitivity, specificity, reproducibility, tissue origin, disease-stage dependence, and treatment responsiveness. Until then, these readouts should be used to support mechanistic interpretation, disease staging, and therapeutic-response assessment, but not as standalone proof of mitochondrial dysfunction.

### 4.2. Biomarker Performance and Limitations

The clinical value of mitochondria-related readouts depends on their context of use. A marker that is useful for mechanistic research may not be suitable for clinical diagnosis, staging, prognosis, or treatment monitoring. Biomarker performance should therefore be evaluated across several dimensions: sensitivity for early mitochondrial stress, specificity for hepatic mitochondria, reproducibility across platforms and laboratories, tissue specificity, disease-stage dependence, vulnerability to confounding factors, and animal-to-human translatability. Current MASLD/MASH guidelines emphasize non-invasive tests mainly for fibrosis risk stratification rather than for diagnosing mitochondrial dysfunction itself. Thus, mitochondria-related readouts should be viewed as supportive mechanistic and translational indicators, not as mature standalone clinical biomarkers [16,187,188].

Acylcarnitines, TCA cycle intermediates, and broader metabolomic signatures may be relatively sensitive to metabolic remodeling, but their specificity is limited. Acylcarnitines can suggest altered fatty acid transport, increased β-oxidation pressure, or incomplete fatty acid oxidation. However, they may also reflect systemic insulin resistance, skeletal muscle metabolism, cardiac metabolism, fasting status, or whole-body substrate availability. Similarly, citrate, succinate, fumarate, malate, α-ketoglutarate, and related intermediates may reflect TCA cycle remodeling, but they are also influenced by amino acid metabolism, anaplerosis, feeding or fasting state, gut-derived metabolites, diabetes, and systemic metabolic stress. Therefore, acylcarnitines are pathway-level readouts of altered fatty acid oxidation, not liver-specific mitochondrial biomarkers [16,174].

Reproducibility is another major limitation. Metabolomics and lipidomics results can differ according to platform and analytical workflow, including LC–MS, GC–MS, NMR, targeted profiling, untargeted profiling, extraction procedures, internal standards, normalization methods, batch effects, and data-processing pipelines. Sample type and handling are also important. Plasma, serum, liver tissue, dried blood spots, and extracellular vesicle-enriched fractions may produce different biological interpretations. Pre-analytical variables such as fasting duration, time of day, storage temperature, freeze–thaw cycles, hemolysis, and delayed processing can alter measured metabolites or lipids. For this reason, candidate biomarkers require standardized sampling, analytical harmonization, external validation, and reproducibility testing before clinical use [16,174,187].

Tissue specificity is particularly important because this review focuses on hepatic mitochondrial dysfunction. Circulating mtDNA can indicate mitochondrial injury or DAMP release, but it is not liver-specific [170,176]. It may originate from hepatocytes, immune cells, platelets, endothelial cells, skeletal muscle, or other injured tissues, and it can be affected by hemolysis, blood-cell lysis, systemic inflammation, and sample preparation. GSH/GSSG and lactate/pyruvate provide useful redox information, but they are also systemic readouts and cannot localize oxidative stress to liver mitochondria. Cardiolipin species are mechanistically closer to mitochondria because cardiolipin is enriched in the inner mitochondrial membrane [180], but cardiolipin-focused lipidomics requires specialized platforms, careful normalization, and tissue- or compartment-aware interpretation. Thus, tissue specificity varies substantially across readouts and should be stated explicitly.

Nutritional state and medication exposure are major confounders. Fasting versus postprandial sampling can change free fatty acids, acylcarnitines, ketone bodies, amino acids, lactate/pyruvate, and TCA-linked metabolites. Exercise, recent weight loss, alcohol exposure, renal function, diabetes control, and acute illness can also alter metabolic readouts. Common MASLD/MASH-related medications may further complicate interpretation. Metformin can affect mitochondrial respiration and AMPK-related metabolism; GLP-1 receptor agonists can reduce body weight and hepatic lipid burden; SGLT2 inhibitors can alter ketone metabolism and substrate use; statins affect lipid metabolism; vitamin E and other antioxidants can change oxidative-stress markers; and resmetirom affects hepatic lipid metabolism and thyroid hormone receptor signaling. These treatments may improve disease biology, but they can also confound biomarker interpretation if sampling conditions are not controlled [187,189].

Stage dependence is a central limitation and also a major reason why single biomarkers perform inconsistently. The same readout may reflect compensation in early disease but failure in advanced disease. For example, increased acylcarnitines in early MASLD may indicate increased FAO pressure or incomplete oxidation during substrate overload. In MASH, lower GSH/GSSG, increased MDA or 4-HNE, and altered lipid peroxide markers may better reflect oxidative injury and antioxidant-buffering failure. In fibrosis, circulating mtDNA or extracellular vesicle-associated mitochondrial cargo may be more closely related to DAMP release, immune activation, and fibrogenesis. In advanced disease, reduced OXPHOS markers, mtDNA instability, or lower mitochondrial biogenesis markers may indicate loss of bioenergetic reserve. Biomarker interpretation therefore requires disease-stage information rather than a single universal cutoff [176,190].

Animal-to-human translation remains a final challenge. Many mitochondria-related markers are derived from diet-induced mouse models, methionine/choline-deficient models, genetic models, or palmitate-treated hepatocyte systems. These models are valuable for mechanism discovery, but they do not fully reproduce human MASLD/MASH heterogeneity. Human disease is shaped by obesity, type 2 diabetes, sex, age, genetic background, diet, medication exposure, alcohol thresholds, immune-cell composition, fibrosis stage, and comorbidities. Animal models also differ in diet composition, feeding duration, fibrosis severity, immune microenvironment, and mitochondrial phenotype. Therefore, animal-derived biomarkers should be validated in human biopsy-linked cohorts, longitudinal studies, and treatment-response settings before being used for clinical decision-making [191,192,193].

Overall, mitochondria-related readouts in MASLD/MASH currently provide mechanistic and translational information rather than definitive clinical diagnosis. Their best use is in multimodal panels that combine clinical phenotype, histology or imaging, fibrosis risk stratification, metabolomics, lipidomics, redox markers, mtDNA-related measures, extracellular vesicle cargo, and treatment response. Future studies should define the intended context of use, standardize sample collection, test analytical reproducibility, evaluate tissue origin, account for nutritional and medication confounders, stratify by disease stage, and validate findings in longitudinal human cohorts. Until these requirements are met, mitochondrial readouts should be presented as supportive indicators of altered metabolic or oxidative stress biology, not as single, mature, or specific biomarkers of hepatic mitochondrial dysfunction.

### 4.3. Therapeutic Strategies Targeting Mitochondrial Stress Across Evidence Levels

**Figure 4:**
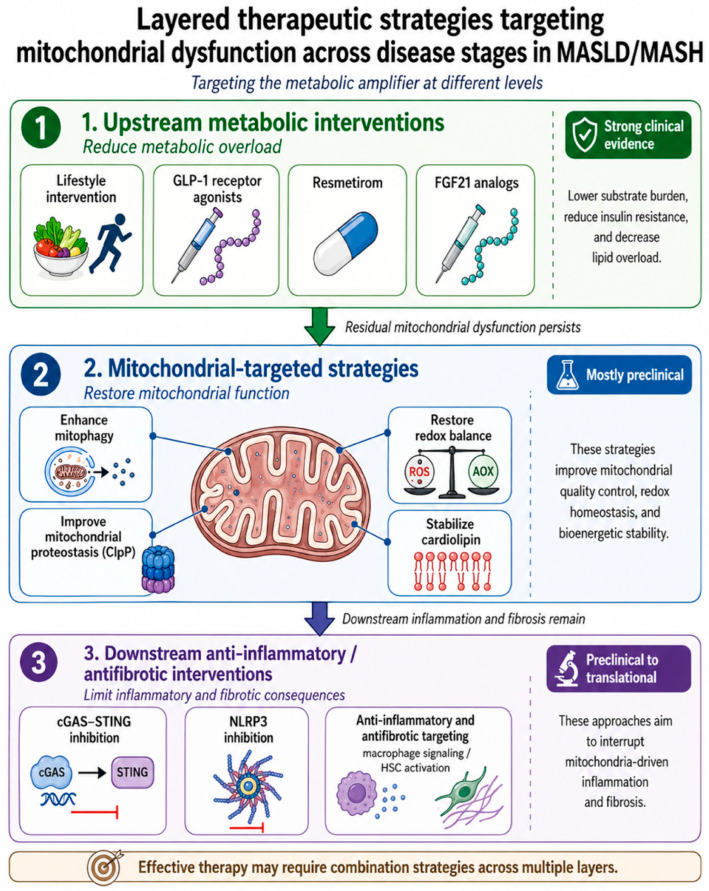
Layered therapeutic strategies targeting mitochondrial dysfunction across disease stages in MASLD/MASH.

Therapeutic strategies related to mitochondrial dysfunction in MASLD/MASH should be organized by evidence strength rather than by whether they appear mechanistically “mitochondrial.” Many clinically supported treatments do not directly repair mitochondria. Instead, they reduce upstream metabolic pressure, including calorie excess, adipose lipolysis, hepatic free fatty acid influx, de novo lipogenesis, insulin resistance, and inflammatory stress. By lowering substrate burden, these interventions may indirectly reduce mitochondrial overload, ROS generation, organelle stress, and downstream inflammatory signaling. In contrast, therapies that directly target mitophagy, cardiolipin, mitochondrial proteostasis, ferroptosis, mitochondrial transfer, or extracellular vesicles remain less clinically mature. This evidence-layered approach is important because mechanistic proximity to mitochondria does not necessarily mean stronger clinical evidence (Figure 4) [188,194]. The evidence hierarchy is summarized in Table 2.

#### 4.3.1. Clinically Supported Metabolic Unloading

Lifestyle intervention remains the foundation of MASLD/MASH treatment. Dietary modification reduces calorie excess and hepatic lipid accumulation, while improved diet quality can reduce insulin resistance and lipotoxic substrate delivery to the liver. Exercise improves systemic insulin sensitivity, skeletal muscle substrate use, and metabolic flexibility, and may improve hepatic mitochondrial oxidative capacity through AMPK-related signaling, enhanced fatty acid oxidation, and muscle–liver crosstalk. These effects are biologically relevant to mitochondrial stress, but they depend on exercise type, intensity, duration, adherence, and baseline metabolic state. Thus, lifestyle and exercise should be viewed as upstream metabolic unloading strategies rather than direct mitochondrial therapies [195,196].

Weight loss provides another clinically supported way to reduce mitochondrial burden. By reducing adipose tissue dysfunction and adipose lipolysis, weight loss decreases circulating free fatty acid delivery to the liver. It also reduces hepatic steatosis, improves insulin resistance, and can improve steatohepatitis; when weight loss is sufficient and sustained, fibrosis may also improve. The mitochondrial benefit is mostly indirect: hepatocytes receive fewer excess substrates, respiratory-chain pressure declines, and lipotoxic redox stress may be reduced. However, durability depends on long-term adherence, maintenance of lean mass, and management of obesity, diabetes, and other cardiometabolic risk factors [195,197].

Incretin-based therapy belongs in this metabolic unloading layer. GLP-1 receptor agonists reduce appetite, body weight, hyperglycemia, insulin resistance, and hepatic fat content. Semaglutide has shown histological benefit in MASH/NASH trials [198] and has also been shown to reduce liver steatosis in NAFLD assessed by magnetic resonance imaging [199]. It is now clinically relevant for selected adults with noncirrhotic MASH and moderate-to-advanced fibrosis following FDA approval [200]. Gastrointestinal adverse events, long-term adherence, lean-mass preservation, and response heterogeneity remain important considerations [198,199,200].

THR-β agonism is another clinically supported metabolic strategy. Thyroid hormone receptor β is enriched in the liver and is involved in hepatic lipid metabolism, cholesterol metabolism, energy expenditure, fatty acid oxidation, and mitochondrial activity [201,202]. Resmetirom is a liver-directed THR-β agonist that improves hepatic lipid handling and reduces lipotoxic pressure [201,202]. It should be described as a liver-directed metabolic therapy with indirect mitochondrial relevance, not as a treatment that directly repairs damaged mitochondria. Its clinical role is strongest in adults with noncirrhotic MASH/NASH and moderate-to-advanced fibrosis, in conjunction with diet and exercise, following FDA approval [203]. Longer-term outcome data will be important for defining effects on clinical events and sustained fibrosis benefit [201].

Overall, lifestyle intervention, weight loss, semaglutide, and resmetirom currently represent the strongest clinical layer. Their shared logic is metabolic unloading. They reduce the substrate and inflammatory burden that drives mitochondrial stress, but they do not specifically normalize mitophagy, cardiolipin integrity, mitochondrial proteostasis, or mtDNA stability. This distinction is important when linking therapeutic benefit to mitochondrial biology.

#### 4.3.2. Emerging Metabolic and Mitochondrial-Modulating Therapies

FGF21-based therapies represent an advanced translational metabolic approach. FGF21 is an endocrine metabolic hormone involved in fasting adaptation, lipid oxidation, energy expenditure, insulin sensitivity, and adipose–liver communication [204]. FGF21 analogs may reduce liver fat, improve adipose tissue dysfunction, lower insulin resistance, and decrease lipotoxic pressure, thereby indirectly supporting mitochondrial flexibility. Pegozafermin and efruxifermin have shown encouraging phase 2 signals in MASH, including improvements in liver fat and histological or fibrosis-related endpoints [205,206]. However, these agents still require larger, longer, and more diverse trials to establish durability, safety, patient selection, and clinical outcome benefit.

Dual and triple incretin therapies are also emerging. GLP-1/GIP dual agonists, GLP-1/glucagon dual agonists, and triple agonists may improve MASH through weight loss, improved insulin sensitivity, reduced hepatic fat, altered substrate use, and increased energy expenditure. Tirzepatide has shown phase 2 benefit for MASH resolution without worsening fibrosis in patients with moderate or severe fibrosis [207], but larger and longer trials are needed to define long-term fibrosis outcomes, safety, and treatment positioning. As with GLP-1 receptor agonists, the mitochondrial benefit of these agents is likely secondary to systemic metabolic improvement rather than direct mitochondrial targeting [207,208].

NAD^+^-related approaches are mechanistically plausible but less clinically established. NAD^+^ links redox balance, sirtuin signaling, mitochondrial substrate oxidation, DNA repair, and mitochondrial adaptation. NAD^+^ depletion or altered NADH/NAD^+^ balance may contribute to metabolic inflexibility and mitochondrial stress in MASLD/MASH. NAD^+^ precursors and strategies that enhance de novo NAD^+^ synthesis or prevent NAD^+^ loss may therefore improve mitochondrial metabolism in experimental systems. However, human MASLD/MASH evidence remains insufficient to present NAD^+^ boosting as an established therapy. Future studies need to define dose, tissue delivery, disease stage, safety, and whether NAD^+^ restoration improves histological or clinical endpoints [209].

Mitochondrial-derived peptides, including MOTS-c, humanin, and SHLPs, are another early translational direction. These peptides may influence insulin sensitivity, oxidative stress, inflammation, mitochondrial adaptation, and cellular stress responses. Their biological rationale is relevant to metabolic liver disease, but MASLD/MASH-specific therapeutic evidence remains early. At present, mitochondrial-derived peptides should be described as experimental or early translational modulators rather than clinically validated treatments [210].

#### 4.3.3. Preclinical Mitochondrial Quality-Control Therapies

Therapies that target mitochondrial quality control are mechanistically closer to mitochondrial dysfunction, but their clinical evidence is weaker. Antioxidant-based strategies aim to reduce ROS, lipid peroxidation, and oxidative stress. Vitamin E has clinical evidence in selected NASH populations, and mitochondria-targeted antioxidants have strong mechanistic rationale. However, ROS also participate in physiological redox signaling, and nonspecific antioxidant suppression may not restore mitochondrial respiration, mitophagy, or metabolic flexibility. Therefore, antioxidant approaches may require careful patient selection, redox phenotyping, and combination with upstream metabolic therapy [40,211].

Cardiolipin-targeting agents are mechanistically attractive because cardiolipin is essential for inner mitochondrial membrane structure, cristae organization, respiratory-chain stability, supercomplex assembly, and cytochrome c interaction. Cardiolipin oxidation or abnormal remodeling can worsen respiratory-chain inefficiency and membrane vulnerability. Agents that stabilize cardiolipin or reduce cardiolipin oxidation could theoretically improve mitochondrial membrane function in MASLD/MASH. However, most evidence remains preclinical or early translational, and cardiolipin remodeling has not yet become a routine therapeutic target or clinical stratification tool in MASH [165].

Mitophagy enhancement is another direct quality-control strategy. Restoring PINK1/Parkin-dependent mitophagy, BNIP3/NIX- or FUNDC1-related mitophagy, AMPK signaling, and autophagy–lysosome function could help remove depolarized, ROS-producing, or mtDNA-damaged mitochondria. Experimental work supports the idea that improving mitochondrial clearance can reduce lipid accumulation, inflammation, and fibrosis-related injury. The limitation is that mitophagy is not simply “more is better.” Excessive or poorly timed mitophagy could reduce mitochondrial mass and impair ATP production. In addition, mitophagic flux is difficult to measure in human liver, making clinical translation challenging [172,180].

Mitochondrial proteostasis is a related therapeutic concept. Respiratory-chain function depends on proper import, folding, assembly, and degradation of mitochondrial proteins. Proteostasis systems involving HSP60, mtHSP70/HSPA9, LONP1, ClpP, and the mitochondrial unfolded protein response may determine whether stressed mitochondria recover or become persistent sources of ROS and DAMPs. Experimental studies suggest that proteostasis failure can worsen mitochondrial dysfunction, inflammation, and fibrosis. However, these targets remain experimental, and pharmacological modulation must avoid excessive protease activation, proteotoxic stress, or impaired respiratory-chain assembly [125].

Ferroptosis-targeting approaches connect mitochondrial stress to lipid-peroxide-driven hepatocyte injury. Potential targets include iron handling, GPX4 insufficiency, glutathione depletion, ACSL4-dependent lipid remodeling, and lipid peroxide accumulation. This approach is mechanistically relevant because MASH involves lipotoxicity, oxidative stress, and lipid peroxidation. However, human evidence is still developing, and ferroptosis should not be described as the dominant or universal form of hepatocyte death in MASH. Ferroptosis-targeting therapy therefore remains a promising but unvalidated strategy requiring human biomarker and treatment-response studies [123].

#### 4.3.4. Speculative Early-Stage Approaches and Combination Strategies

Mitochondrial transplantation is a highly speculative strategy for MASLD/MASH. In theory, delivery of functional mitochondria could improve bioenergetic reserve or replace severely damaged organelles. In practice, major barriers remain, including delivery to the liver, cellular uptake, organelle survival, immune response, tissue targeting, durability, scalability, manufacturing, and safety. For MASLD/MASH, mitochondrial transplantation should therefore be mentioned only as an early conceptual strategy rather than a near-term clinical therapy [212].

Extracellular vesicle-based therapy is similarly promising but complex. EVs can transfer miRNAs, proteins, lipids, mitochondrial signals, and immunomodulatory cargo. They may be engineered to reduce hepatocyte injury, macrophage inflammation, or HSC activation. However, EVs can also propagate harmful DAMPs, oxidized lipids, or profibrotic signals. Their therapeutic development is limited by heterogeneity, cargo definition, biodistribution, dosing, manufacturing reproducibility, off-target effects, and safety. EV-based approaches therefore remain early-stage and require rigorous functional characterization before clinical translation [213].

Because MASLD/MASH is a multi-hit and multicellular disease, combination therapy may ultimately be more realistic than single-pathway intervention. Potential combinations include metabolic unloading plus THR-β agonism, incretin-based therapy plus FGF21 analogs, metabolic therapy plus antifibrotic therapy, or metabolic therapy plus mitochondrial quality-control support. This logic is attractive because lipid overload, insulin resistance, mitochondrial inefficiency, inflammation, and fibrosis reinforce one another. However, combination therapy introduces new challenges, including safety, drug–drug interactions, cost, adherence, endpoint selection, and patient stratification. Future trials should therefore match therapies to disease stage and dominant biology rather than simply combining agents empirically [214,215].

In summary, the therapeutic implications of mitochondrial dysfunction in MASLD/MASH should be interpreted across evidence layers. Clinically supported therapies mainly reduce upstream metabolic pressure and indirectly relieve mitochondrial burden. Emerging metabolic therapies may improve mitochondrial flexibility through systemic metabolic remodeling. Direct mitochondrial quality-control therapies are mechanistically appealing but remain mostly preclinical. Organelle replacement and EV-based strategies are still speculative. This evidence-based hierarchy prevents overstatement and connects mitochondrial biology to realistic therapeutic translation.

### 4.4. Patient Stratification, Treatment-Response Prediction, and Translational Barriers

Patient stratification is essential because MASLD/MASH is highly heterogeneous. Mitochondrial dysfunction may act as a common amplifier of disease progression, but the upstream drivers and downstream consequences differ across patients. In some individuals, obesity and adipose insulin resistance dominate, leading to increased free fatty acid flux to the liver. In others, type 2 diabetes, hyperinsulinemia, hyperglycemia, and de novo lipogenesis are stronger drivers. Some patients may have a stronger genetic contribution, whereas others have already progressed to a fibrosis-dominant stage in which inflammatory and fibrogenic pathways may be more important than steatosis itself. Therefore, one mitochondrial-targeted therapy is unlikely to be appropriate for all patients. Stratification should define the dominant disease driver, mitochondrial phenotype, fibrosis risk, and therapeutic goal before selecting a treatment strategy [22,58,188,216].

Disease stage and fibrosis stage should be the first layer of stratification. In early MASLD or simple steatosis, the dominant abnormalities are usually lipid overload, insulin resistance, and partial mitochondrial compensation. These patients may benefit most from lifestyle intervention, exercise, weight loss, GLP-1 receptor agonists, and other metabolic unloading strategies, rather than from aggressive mitochondrial quality-control therapy. In MASH, mitochondrial stress is more likely to be injurious, with ROS generation, ER stress, lipid peroxidation, inflammatory signaling, and impaired mitophagy. This stage may require metabolic unloading together with anti-inflammatory, redox-modulating, or mitochondrial quality-control approaches. In F2–F3 fibrosis, prognosis becomes more strongly determined by fibrotic remodeling, hepatic stellate cell activation, macrophage–HSC crosstalk, and ECM deposition; therefore, clinical trials and therapeutic endpoints should include fibrosis improvement and antifibrotic readouts. In cirrhosis or advanced disease, hepatic bioenergetic reserve and regenerative capacity are reduced, and drug safety becomes more important. Thus, disease stage determines whether mitochondrial dysfunction is mainly compensatory, injurious, inflammatory, fibrogenic, or associated with loss of regenerative reserve [113].

Metabolic phenotype is another key determinant of mitochondrial stress and treatment response. In obesity-dominant MASLD/MASH, adipose tissue dysfunction and increased lipolysis increase FFA delivery to the liver, making weight-loss therapies and adipose-liver metabolic unloading especially relevant. In T2DM- or insulin resistance-dominant disease, hyperinsulinemia, hyperglycemia, de novo lipogenesis, impaired substrate switching, and altered adipose–liver communication may increase mitochondrial substrate pressure and reduce metabolic flexibility. These patients may be more likely to benefit from GLP-1 receptor agonists, dual incretins, SGLT2-related metabolic therapies, or other interventions that improve glycemic control and substrate use. Lean MASLD should not be explained only by excess weight or calorie overload. In such patients, genetic susceptibility, lipid-droplet biology, VLDL export, and phospholipid remodeling may have greater relative importance. Thus, metabolic phenotype affects both the pattern of mitochondrial stress and the likely response to metabolic unloading or mitochondrial-modulating therapy [58,216,217].

Demographic and ancestry-related heterogeneity should also be incorporated. Sex hormones influence fat distribution, insulin sensitivity, inflammatory tone, and mitochondrial metabolism, and the risk profile may differ between men, premenopausal women, and postmenopausal women. Aging is associated with mtDNA damage, reduced mitochondrial biogenesis, impaired mitophagy, oxidative stress, and lower regenerative reserve, which may shift older patients toward mitochondrial quality-control failure while also increasing safety concerns related to polypharmacy and comorbidity. Ethnicity and ancestry may influence MASLD/MASH risk through differences in genetic variants, visceral adiposity, diabetes prevalence, lipid handling, socioeconomic exposures, and access to care. Biomarkers and treatment responses should therefore not be assumed to perform identically across sex, age, and ancestry groups. Future trials should include prespecified subgroup analyses rather than treating demographic heterogeneity as background noise [218,219,220].

Genetic variants may define subgroups with different lipid-handling defects, fibrosis risk, and treatment responsiveness. PNPLA3 variants are linked to hepatic fat accumulation, inflammation, and fibrosis risk [221], probably through altered lipid-droplet remodeling. TM6SF2 variants affect VLDL secretion and hepatic lipid retention [222], connecting genetic susceptibility to impaired lipid export. MBOAT7 variants are associated with phospholipid remodeling and may influence hepatic inflammation and fibrosis [223]. HSD17B13 loss-of-function variants appear protective against liver injury and may modify progression risk or therapeutic targeting [224]. GCKR variants connect glucose and lipid metabolism with de novo lipogenesis and may interact with diabetic metabolic status. These variants should not be used as single deterministic markers, but they can help explain why patients with similar BMI or liver fat may have different mitochondrial phenotypes, fibrosis risk, and treatment responses [217,218,221,222,223,224].

Molecular biomarkers may help refine stratification, but most remain exploratory. Future stratification should not rely only on BMI, ALT, histology, or imaging. A more informative approach may combine fibrosis stage with metabolomics, acylcarnitines, TCA intermediates, GSH/GSSG, lipidomics, cardiolipin species [220], mtDNA copy number, circulating mtDNA, inflammatory cytokines, EV cargo, and genetic variants. These readouts could help identify patients dominated by lipid overload, redox stress, mtDNA instability, inflammatory DAMP release, or fibrogenic signaling. However, current biomarkers often lack sufficient validation for sensitivity, specificity, reproducibility, tissue origin, disease-stage dependence, and treatment-response prediction. Therefore, biomarkers should be used to enrich mechanistic subgroups and support therapeutic-response assessment, but most are not yet ready to guide clinical decisions as standalone tests [16,130,225].

Safety and translational barriers must be considered because mitochondrial pathways are fundamental to whole-body metabolism. Excessive antioxidant therapy may interfere with physiological redox signaling. Mitophagy enhancers may remove damaged mitochondria, but excessive or poorly timed mitophagy could reduce mitochondrial mass and compromise ATP production. NAD^+^ boosters may have broad metabolic effects beyond the liver. THR-β therapy requires attention to thyroid-axis-related and lipid-metabolic effects, incretin therapies can cause gastrointestinal adverse events and excessive weight loss in susceptible patients, and antifibrotic or EV-based therapies require long-term safety assessment. Preclinical-to-clinical translation is another major barrier. Diet-induced mouse models, MCD models, genetic models, organoids, and palmitate-treated hepatocytes are useful for mechanism discovery, but they do not fully reproduce human disease duration, fibrosis severity, immune microenvironment, metabolic comorbidities, sex and age effects, medication exposure, or genetic diversity. Therefore, preclinical mitochondrial benefit should not be assumed to predict clinical histological benefit. Future trials should combine stage-based enrollment, metabolic phenotype, genetic background, mechanistic biomarkers, safety monitoring, and clinically meaningful endpoints.

## 5. Conclusions and Future Perspectives

### 5.1. Mitochondrial Dysfunction as a Stage-Dependent Amplifier Rather than a Single Lesion

Mitochondrial dysfunction is best understood as an active participant in MASLD/MASH progression, not simply as a consequence of lipid accumulation. Excess free fatty acids and lipotoxic species place sustained pressure on hepatocytes, and this pressure can disturb fatty acid oxidation, redox balance, ER-mitochondria communication, mitochondrial dynamics, mitophagy, and danger-signal release. These changes help link metabolic overload to inflammation and fibrotic remodeling [17,165,169,226,227].

The main challenge for future work is to define timing and context. Mitochondrial oxidation may be compensatory in early disease but maladaptive when substrate pressure persists. ROS, ER stress, mitophagy failure, inflammation, and fibrosis should therefore be studied as interacting processes that change over time, rather than as separate endpoints.

This perspective also affects therapy. Interventions that reduce metabolic overload currently have the strongest clinical support, while direct mitochondrial restoration remains biologically plausible but less clinically mature. Better stage-specific and cell-type-specific studies will be needed before mitochondria-centered strategies can be matched to patient subgroups.

### 5.2. Unresolved Questions in Causality, Timing, and Cell Specificity

Mitochondrial dysfunction is frequently described as a driver of MASLD/MASH progression, but the available evidence does not establish it as a universal initiating event. Lipid overload, insulin resistance, type 2 diabetes, ER stress, inflammatory cytokines, hypoxia, and tissue remodeling can all impair mitochondrial metabolism. Therefore, mitochondrial dysfunction should not be interpreted as a single upstream cause in all patients or all disease stages [228]. A more cautious interpretation is that mitochondrial dysfunction may function as a cause, consequence, or amplifier depending on disease stage, metabolic context, and cell type [164,229].

Several lines of evidence support an active driver role for mitochondrial dysfunction once adaptive capacity is exceeded. Increased fatty acid oxidation and oxidative phosphorylation can initially help hepatocytes handle excess lipid input, but persistent substrate pressure can increase respiratory-chain stress, electron leak, ROS generation, and lipid peroxidation [127,129]. Genetic or molecular disruption of mitochondrial function can also aggravate steatohepatitis in experimental systems. For example, impairment of mitochondrial complex I-related function can aggravate progression toward NASH [230], while altered mitochondrial stress signaling or RNA-processing pathways may also worsen lipid accumulation, hepatocyte injury, and inflammatory progression [228]. In addition, damaged mitochondria can release mtDNA, cardiolipin, mitochondrial RNA, ATP, and other DAMPs that activate cGAS–STING, TLR9, AIM2, or NLRP3-related pathways [127,128,129]. When mitophagy fails, damaged mitochondria persist and continue to generate ROS and danger signals. Through extracellular vesicles and DAMP-mediated communication, mitochondrial stress in hepatocytes can influence Kupffer cells, recruited macrophages, hepatic stellate cells, and fibrogenic remodeling [143,144,145,146,147,158]. Thus, mitochondrial dysfunction can actively drive disease progression once mitochondrial stress shifts from compensation to maladaptation.

At the same time, mitochondrial dysfunction may also be a downstream consequence of chronic metabolic and inflammatory stress. Obesity, adipose insulin resistance, type 2 diabetes, increased adipose lipolysis, hepatic free fatty acid influx, and de novo lipogenesis can precede overt mitochondrial failure [229,231]. Lipotoxic species such as diacylglycerols, ceramides, saturated fatty acids, palmitate, and cholesterol can accumulate before severe respiratory impairment is detected. ER stress, calcium overload, cytokine exposure, hypoxia, ECM stiffness, and impaired nutrient or oxygen diffusion can further damage mitochondria during later disease. Longitudinal and model-based studies also show that the timing of mitochondrial impairment is not uniform: some models show increased or preserved mitochondrial oxidation in early steatosis, whereas others show impaired respiration after steatohepatitis or fibrosis has already developed [232,233]. Therefore, mitochondrial dysfunction may also represent a downstream response to chronic lipid excess, inflammatory stress, and tissue remodeling rather than the earliest pathogenic event.

The timing of mitochondrial dysfunction is likely stage dependent. In early MASLD or simple steatosis, mitochondria may be in a compensatory state, with fatty acid oxidation and oxidative phosphorylation preserved or increased and metabolic flexibility only partially reduced. This stage should not automatically be labeled as mitochondrial failure. In MASH, persistent lipid overload and inflammatory stress can promote compensation failure, inefficient respiration, ROS generation, lipid peroxidation, ER–mitochondria stress, and impaired mitophagy. At this stage, mitochondrial dysfunction becomes a stronger amplifier of hepatocyte injury and sterile inflammation. During fibrosis, mitochondrial DAMP release and EV-mediated signaling may support macrophage activation, hepatic stellate cell activation, and ECM deposition. In cirrhosis or advanced disease, severe respiratory impairment, mtDNA instability, reduced biogenesis, impaired quality control, and poor regenerative reserve may make mitochondrial dysfunction more important for disease maintenance than disease initiation. Thus, the role of mitochondrial dysfunction may shift from adaptation to amplification and finally to loss of bioenergetic reserve [69,164,232,233].

Cell specificity is another unresolved issue. Hepatocyte mitochondria are central to lipid handling, fatty acid oxidation, OXPHOS, ROS generation, mtDNA damage, and DAMP release. In Kupffer cells and recruited macrophages, mitochondrial metabolism can shape immune activation, inflammasome signaling, cytokine release, and responses to hepatocyte-derived mtDNA or EV cargo. In hepatic stellate cells, mitochondrial remodeling and ROS may contribute to activation, collagen production, and the maintenance of a fibrogenic phenotype. Neutrophils, T cells, NK cells, NKT cells, dendritic cells, and B cells may further amplify inflammation through immune-cell metabolism, cytokine production, hepatocyte killing, and fibrosis modulation. Liver sinusoidal endothelial cells may also contribute through vascular stress, capillarization, endothelial dysfunction, and inflammatory recruitment. Therefore, mitochondrial dysfunction is not identical across liver cell populations. Hepatocyte mitochondrial stress may initiate metabolic injury, whereas immune and stromal cell mitochondrial remodeling may shape inflammation, fibrogenesis, and disease persistence [22,127,129].

Future studies should define when mitochondrial dysfunction appears, in which cell type it occurs, and whether correcting it changes disease trajectory. Longitudinal human studies are needed to distinguish compensation from failure and to determine whether mitochondrial changes precede, accompany, or follow inflammation and fibrosis. Stage-resolved liver biopsy studies, non-invasive metabolic readouts, isotope tracing, single-cell and spatial transcriptomics, cell-type-specific mitochondrial assays, organoid systems, liver-on-chip models, and multicellular co-culture systems may help clarify causality. Animal and hepatocyte-only models remain useful for mechanism discovery, but their findings require human validation because human MASLD/MASH is shaped by obesity, type 2 diabetes, genetic risk, sex, age, ethnicity, medication exposure, immune heterogeneity, and fibrosis stage. Future trials should test whether mitochondrial biomarkers can distinguish adaptive remodeling from mitochondrial failure and whether mitochondrial-targeted interventions improve clinically meaningful outcomes in the patient subgroup most likely to benefit.

### 5.3. Future Therapeutic Perspectives and Patient Stratification

Future therapeutic strategies for MASLD/MASH should move beyond single-pathway intervention. Mitochondrial dysfunction is not an isolated therapeutic target; it interacts with lipid overload, insulin resistance, ER stress, oxidative injury, inflammation, and fibrosis. Therefore, a single mitochondrial drug is unlikely to benefit all patients or all disease stages. A more realistic direction is stage-specific, phenotype-specific, and combination-based therapy. In this framework, mitochondrial dysfunction is treated as a disease amplifier whose therapeutic relevance depends on the dominant upstream driver and downstream consequence in each patient, rather than as a uniform disorder shared by all MASLD/MASH cases [188,229].

Stage-specific therapy will be central to future treatment design. In early MASLD or simple steatosis, the main therapeutic goal is metabolic unloading. Lifestyle intervention, exercise, weight loss, GLP-1 receptor agonists, dual incretins, THR-β agonism, and other metabolic approaches may reduce hepatic lipid input, improve insulin resistance, and lower mitochondrial substrate pressure. At this stage, mitochondria may still be partly compensatory, so aggressive mitochondrial repair therapy may not be necessary. In MASH, persistent lipid overload and inflammatory injury are more prominent; treatment may need to combine metabolic unloading with injury-control strategies targeting ROS, lipid peroxidation, ER stress, defective mitophagy, or ferroptosis-related hepatocyte death. In fibrosis-dominant disease, therapy should increasingly focus on the inflammation–fibrosis axis, including macrophage–HSC crosstalk, TGF-β signaling, EV-mediated communication, and ECM deposition. In cirrhosis or advanced disease, reduced mitochondrial reserve and impaired regenerative capacity narrow the therapeutic safety window; direct mitochondrial stimulation or strong metabolic manipulation may require particularly careful endpoint selection and safety monitoring [22,234].

Combination therapy may better match the multi-hit nature of MASLD/MASH than monotherapy. No single intervention is likely to simultaneously correct obesity, insulin resistance, lipotoxicity, oxidative stress, inflammation, fibrosis, and mitochondrial quality-control failure. Rational combinations may include metabolic unloading plus THR-β agonism to reduce body weight and improve hepatic lipid metabolism; GLP-1 receptor agonists or dual incretins plus FGF21 analogs to improve weight, insulin sensitivity, adipose function, and lipid handling; metabolic therapy plus antifibrotic therapy for F2–F3 or fibrosis-dominant patients; or metabolic therapy plus mitochondrial quality-control support in patients with evidence of ROS excess, mitophagy failure, mtDNA injury, or lipid-peroxide stress. However, combination therapy is not yet a mature solution. Future studies need to define optimal sequencing, dosing, safety, drug–drug interactions, cost-effectiveness, and mechanism-based endpoints before such strategies can be broadly implemented [194,235].

Biomarker-guided therapy will be needed to connect mitochondrial biology with clinical decision-making. Future trials should not rely only on ALT, BMI, liver fat content, or histology. Mitochondria-related readouts such as acylcarnitines, TCA cycle intermediates, GSH/GSSG, lipid peroxidation products, cardiolipin species, mtDNA copy number, circulating mtDNA, EV-associated mitochondrial cargo, and metabolomic or lipidomic panels may help identify patients with dominant lipid overload, redox imbalance, mitochondrial membrane stress, DAMP release, or quality-control failure. These biomarkers could enrich mechanistic subgroups, monitor target engagement, and predict treatment response. However, no single mitochondrial biomarker is currently mature enough for standalone clinical decision-making. Future biomarker panels must be validated for sensitivity, specificity, reproducibility, tissue origin, disease-stage dependence, longitudinal change, and treatment-response prediction [16].

Patient stratification should integrate clinical, metabolic, genetic, and molecular information. Patients with obesity- or T2DM-dominant disease may be more likely to benefit from weight loss, GLP-1 receptor agonists, dual or triple incretins, and broader metabolic unloading. Patients with lipid-handling or genetic-risk profiles may require different interpretation, particularly when variants such as PNPLA3, TM6SF2, MBOAT7, GCKR, or HSD17B13 influence lipid-droplet remodeling, VLDL secretion, phospholipid remodeling, glucose–lipid coupling, or susceptibility to liver injury. Fibrosis-dominant patients require antifibrotic endpoints, HSC/macrophage-focused strategies, combination therapy, and long-term outcome monitoring. Patients with high mitochondrial injury biomarkers may eventually be candidates for antioxidant, cardiolipin-targeting, mitophagy-enhancing, NAD^+^-restoring, or ferroptosis-modulating approaches. Sex, age, ethnicity, medication exposure, and comorbidities should also be included because they may affect mitochondrial phenotype, safety, and therapeutic response [217,218].

Finally, direct mitochondrial therapy faces important translational barriers. Mitochondria are essential in many tissues, so off-target effects may occur outside the liver. ROS and mitophagy are double-edged processes: ROS participate in physiological signaling, and mitophagy is protective only when appropriately timed and balanced. Human validation is also difficult because liver biopsy is invasive, mitochondrial flux is hard to measure in patients, and many human samples are cross-sectional. Animal models and palmitate-treated hepatocyte systems remain useful for mechanism discovery, but they cannot fully reproduce human obesity, T2DM, fibrosis duration, immune heterogeneity, genetic diversity, medication exposure, or multicellular liver remodeling. Therefore, the future of mitochondrial therapy in MASLD/MASH depends less on finding one universal mitochondrial drug and more on matching mechanism, disease stage, biomarker profile, and patient phenotype in longitudinal human studies with clinically meaningful endpoints [16,191].

### 5.4. MOMP/miMOMP as a Cautious Emerging Hypothesis

Mitochondrial outer membrane permeabilization (MOMP) may provide a useful conceptual framework for understanding mitochondrial content release in MASLD/MASH, but it should be presented as a cautious and emerging hypothesis rather than as an established disease mechanism. Direct evidence for sublethal MOMP in metabolically stressed hepatocytes remains limited. Nevertheless, studies of hepatocyte injury and liver stress suggest that oxidative stress, lipid stress, mitochondrial glutathione depletion, cardiolipin peroxidation, and Bax-related signaling can increase mitochondrial membrane vulnerability and promote mitochondrial content leakage [236,237]. These observations make MOMP biologically plausible in MASLD/MASH, but they do not yet prove that MOMP is a frequent or causal event in human disease.

This hypothesis is relevant because mitochondrial content release does not necessarily require immediate complete apoptosis. Recent work in non-MASLD contexts has shown that transient lysosomal damage can trigger sublethal MOMP through BAK/BAX macropores and promote mtDNA release with downstream cGAS signaling. Although this mechanism has not been directly demonstrated in MASLD/MASH, it provides a possible explanation for how stressed but still viable hepatocytes might sustain sterile inflammatory signaling. This concept is also compatible with MASLD/MASH studies showing that mitochondrial DNA can contribute to innate immune activation, including AIM2 inflammasome activation and hepatocyte pyroptosis [134,238].

A related concept is minority mitochondrial outer membrane permeabilization (miMOMP), in which only a subset of mitochondria undergoes outer membrane permeabilization after sublethal stress. In other biological contexts, miMOMP has been linked to DNA damage, genome instability, and chronic stress signaling without immediate cell death [239]. For MASLD/MASH, this remains speculative but potentially important. Metabolically stressed hepatocytes might experience limited mitochondrial membrane injury that permits mitochondrial content release while avoiding full apoptotic collapse. Such a process could help explain persistent mtDNA sensing, low-grade inflammatory signaling, hepatocyte senescence, and fibrogenic remodeling during chronic disease progression.

Current evidence is not strong enough to define MOMP or miMOMP as core mechanisms of MASLD/MASH. They are better framed as testable hypotheses that may connect chronic mitochondrial membrane stress with mtDNA release, innate immune activation, and fibrogenic signaling. Future studies should determine whether sublethal MOMP or miMOMP occurs during lipotoxic or oxidative stress and ER–lysosome–mitochondria stress in hepatocytes; whether it differs across simple steatosis, MASH, fibrosis, and cirrhosis; and whether blocking inappropriate mitochondrial membrane permeabilization can reduce inflammatory or fibrogenic progression without impairing necessary cell-death or quality-control responses.

## Figures and Tables

**Table 1:** Evidence types and translational interpretation of mitochondrial mechanisms in MASLD/MASH.

Mechanistic Domain	Human Evidence	Animal Evidence	In Vitro Evidence	Translational Interpretation
Lipid overload and substrate pressure	Associations with obesity, insulin resistance, T2DM, hepatic fat, and metabolic phenotype.	Diet-induced and genetic models link lipid overload to FAO, ETC pressure, steatohepatitis, and fibrosis.	Palmitate-, oleate-, FFA-, glucose-, or fructose-loaded hepatocytes model substrate overload and lipotoxic stress.	Strong upstream relevance, but timing and causality require longitudinal and flux-based human validation.
Bioenergetic remodeling	Human studies suggest preserved/increased oxidation early and impaired respiration in advanced disease.	Models show stage- and diet-dependent changes in FAO, TCA flux, OXPHOS, ATP, and coupling.	Assays test respiration, ATP, membrane potential, substrate oxidation, and respiratory stress.	Supports a transition from compensation to inefficient respiration, but timing varies by model and stage.
ETC dysfunction, ROS, and lipid peroxidation	Human markers support oxidative stress and altered mitochondrial function, but source specificity is limited.	Models show mitochondrial ROS, lipid peroxidation, cardiolipin vulnerability, and injury amplification.	Lipotoxic stress increases ROS, lowers membrane potential, and damages proteins, lipids, and mtDNA.	ROS readouts require functional context and should not be treated as mitochondria-specific alone.
Organelle crosstalk	Human evidence is mostly indirect through tissue markers, omics signatures, and disease severity.	MAM remodeling, Ca^2+^ stress, lysosomal dysfunction, defective lipophagy, and peroxisomal stress interact with mitochondria.	Models test Ca^2+^ transfer, ER stress, lysosomal acidification, autophagic flux, and mitochondrial injury.	Biologically plausible and experimentally supported, but temporal human evidence remains limited.
Dynamics, mitophagy, and autophagy markers	Human studies report altered turnover, morphology, and quality-control markers, but static markers do not prove flux.	DRP1, MFN1/2, OPA1, PINK1/Parkin, BNIP3/NIX, FUNDC1, Beclin-1, LC3-II, p62, ATG5/ATG7, and lysosomal pathways are implicated.	Direct manipulation of fission, fusion, mitophagy, autophagosome formation, and lysosomal degradation is possible.	Interpretation requires phosphorylation-site specificity, autophagic flux, lysosomal degradation, and disease-stage context.
Biogenesis, mtDNA maintenance, and UPRmt	Includes mtDNA copy number, mitochondrial gene expression, and biopsy-linked mitochondrial markers.	PGC-1alpha, NRF1, TFAM, mtDNA maintenance, respiratory remodeling, and proteostasis are linked to adaptation or failure.	UPRmt-related HSP60, HSP90, mtHSP70/HSPA9, LONP1, and ClpP can be tested under lipotoxic stress.	Biogenesis and UPRmt markers may distinguish adaptation from failure, but are not standalone functional proof.
Mitochondrial DAMPs, EV cargo, and inflammation	Circulating mtDNA, inflammatory markers, fibrosis, and severity are associated, but cellular origin is uncertain.	mtDNA, mtRNA, cardiolipin, ATP, HMGB1, and EV cargo activate TLR9, cGAS-STING, AIM2, or NLRP3-related pathways.	Co-culture models test hepatocyte-macrophage and hepatocyte-stellate-cell communication.	Danger signaling is a plausible amplifier of inflammation and fibrosis; cell specificity needs single-cell and spatial validation.
Gut-liver-mitochondria axis	Gut dysbiosis, gut-derived metabolites, bile acids, and metabolic phenotype are linked with MASLD/MASH risk.	Microbiota-derived metabolites, endotoxin-related signals, and bile acid changes alter hepatic metabolism and mitochondrial stress.	Microbial metabolites, bile acids, and inflammatory mediators can be tested in hepatocyte systems.	This axis should be framed as a bidirectional metabolic and inflammatory network rather than a single linear pathway.
Therapeutic translation	Strongest evidence supports metabolic unloading, weight loss, incretin-based therapy, and THR-beta agonism.	Models support quality-control, redox, mitophagy, ferroptosis, and antifibrotic interventions.	Cell models support target discovery but cannot represent multicellular MASH or fibrosis.	Separate clinically supported strategies from preclinical mitochondrial hypotheses and prioritize stage-specific trials.

**Table 2:** Evidence levels of therapeutic strategies targeting mitochondrial dysfunction in MASLD/MASH.

Therapeutic Strategy	Main Layer	Representative Agents/Directions	Evidence Level	Direct Mitochondrial Targeting?
Lifestyle intervention	Upstream metabolic unloading	Weight loss, diet, exercise	Strong clinical	No (indirect)
THR-β agonism	Upstream metabolic unloading	Resmetirom	Strong clinical	No (indirect, liver-directed metabolic effect)
Incretin-based therapy	Upstream metabolic unloading	Semaglutide (Wegovy); GLP-1 receptor agonists; dual/triple incretins	Strong clinical for semaglutide; growing clinical for other incretin therapies	No (mainly indirect)
FGF21-based therapy	Advanced translational metabolic	Efruxifermin and related FGF21 analogs	Early-to-mid clinical	Mostly indirect
MGAT2 inhibition	Early translational metabolic	MGAT2 inhibitors	Preclinical + phase 1 obesity data	No (indirect)
Mitochondrial homeostasis/mitophagy	Midstream mitochondrial maintenance	Mitophagy restoration; ClpP; PINK1/Parkin-related strategies	Mostly preclinical	Yes
Downstream inflammatory/fibrotic interruption	Downstream consequence control	GLP-1/GLP-2 dual agonism and related anti-inflammatory/antifibrotic strategies	Mostly preclinical	Usually indirect

## Data Availability

No new data were created or analyzed in this study. Data sharing is not applicable to this article.

## Abbreviations

CPT1, carnitine palmitoyl transferase 1; ER, endoplasmic reticulum; FAO, fatty acid oxidation; FGF21, fibroblast growth factor 21; GLP-1, glucagon-like peptide-1; GSH/GSSG, reduced/oxidized glutathione; HSC, hepatic stellate cell; MAM, mitochondria-associated membrane; MASLD, metabolic-dysfunction-associated steatotic liver disease; MASH, metabolic-dysfunction-associated steatohepatitis; miMOMP, minority mitochondrial outer membrane permeabilization; MOMP, mitochondrial outer membrane permeabilization; mtDNA, mitochondrial DNA; OXPHOS, oxidative phosphorylation; ROS, reactive oxygen species; STING, stimulator of interferon genes; TCA, tricarboxylic acid; THR-β, thyroid hormone receptor beta; cGAS, cyclic GMP–AMP synthase; UPRmt, mitochondrial unfolded protein response; DAMPs, damage-associated molecular patterns; EVs, extracellular vesicles; ETC, electron transport chain.
